# Cooperative Control of Ecdysone Biosynthesis in *Drosophila* by Transcription Factors Séance, Ouija Board, and Molting Defective

**DOI:** 10.1534/genetics.117.300268

**Published:** 2017-11-29

**Authors:** Outa Uryu, Qiuxiang Ou, Tatsuya Komura-Kawa, Takumi Kamiyama, Masatoshi Iga, Monika Syrzycka, Keiko Hirota, Hiroshi Kataoka, Barry M. Honda, Kirst King-Jones, Ryusuke Niwa

**Affiliations:** * Faculty of Life and Environmental Sciences, University of Tsukuba, 305-8572, Ibaraki, Japan; † Department of Biological Sciences, University of Alberta, Edmonton, Alberta T6G 2E9, Canada; ‡ Graduate School of Life and Environmental Sciences, University of Tsukuba, 305-8572, Ibaraki, Japan; § Department of Integrated Biosciences, Graduate School of Frontier Sciences, The University of Tokyo, Kashiwa, Chiba 277-8562, Japan; ** Department of Molecular Biology and Biochemistry, Simon Fraser University, Burnaby, British Columbia V5A 1S6, Canada; †† Precursory Research for Embryonic Science and Technology, Japan Science and Technology Agency, Kawaguchi, Saitama 332-0012, Japan

**Keywords:** *Drosophila*, ecdysone, heterochromatin, steroid hormone biosynthesis, transcription, zinc finger

## Abstract

Ecdysteroids are steroid hormones that control many aspects of development and physiology. During larval development, ecdysone is synthesized in an endocrine organ called the prothoracic gland through a series of ecdysteroidogenic enzymes encoded by the Halloween genes. The expression of the Halloween genes is highly restricted and dynamic, indicating that their spatiotemporal regulation is mediated by their tight transcriptional control. In this study, we report that three zinc finger-associated domain (ZAD)-C_2_H_2_ zinc finger transcription factors—Séance (Séan), Ouija board (Ouib), and Molting defective (Mld)—cooperatively control ecdysone biosynthesis in the fruit fly *Drosophila melanogaster*. Séan and Ouib act in cooperation with Mld to positively regulate the transcription of *neverland* and *spookier*, respectively, two Halloween genes. Remarkably, loss-of-function mutations in *séan*, *ouib*, or *mld* can be rescued by the expression of *neverland*, *spookier*, or both, respectively. These results suggest that the three transcription factors have distinct roles in coordinating the expression of just two genes in *Drosophila*. Given that *neverland* and *spookier* are located in constitutive heterochromatin, Séan, Ouib, and Mld represent the first example of a transcription factor subset that regulates genes located in constitutive heterochromatin.

IN insects, ecdysteroids are the principal steroid hormones that control many aspects of development and physiology, including molting, metamorphosis, longevity, and neuronal functions (Ishimoto and Kitamoto 2011; Yamanaka *et al.* 2013; Niwa and Niwa 2014; Uryu *et al.* 2015). Among endogenously identified ecdysteroids, including 20-deoxymakisterone A and 24(28)-dehydromakisterone A (Lavrynenko *et al.* 2015), the best-characterized biologically active ecdysteroid is 20-hydroxyecdysone (20E), which is derived from the prohormone ecdysone.

Similar to vertebrate steroid hormones, 20E is synthesized *in vivo* through a series of enzymatic steps from suitable sterol precursors such as cholesterol. Although ecdysteroidogenic genes have been intensively studied over the last 15 years, the ecdysone biosynthetic pathway is still not completely understood (Rewitz *et al.* 2006; Niwa and Niwa 2014). During larval development, ecdysone is synthesized in an endocrine organ called the prothoracic gland (PG), whereas the conversion of ecdysone to 20E occurs in peripheral tissues via the cytochrome P450 monooxygenase Shade (Shd) (Petryk *et al.* 2003; Yamanaka *et al.* 2013). In the first step toward ecdysone synthesis in the PG, cholesterol is converted to 7-dehydrocholesterol (7DC) by the Rieske oxygenase Neverland (Nvd) (Yoshiyama *et al.* 2006; Yoshiyama-Yanagawa *et al.* 2011). Although the intermediate steps that convert 7DC to 5β-ketodiol are not entirely understood (Ono *et al.* 2012; Saito *et al.* 2016), at least three enzymes are thought to be involved in this conversion, including Shroud (Sro) (Niwa *et al.* 2010), Spook/Spookier (Spok) (Namiki *et al.* 2005; Ono *et al.* 2006), and CYP6T3 (Ou *et al.* 2011). The conversion from 5β-ketodiol to ecdysone is subsequently catalyzed by three P450 enzymes (Warren *et al.* 2002, 2004; Niwa *et al.* 2004, 2005). We define here “Halloween genes” collectively as genes encoding enzymes involved in the conversion of dietary sterols to ecdysteroids. Null mutations in most of the Halloween genes (except *nvd*, *spok*, and *Cyp6t3*) cause characteristic embryonic phenotypes, where a deficiency in ecdysteroids causes the cuticle to remain undifferentiated (Rewitz *et al.* 2006).

The temporal profiles of the Halloween genes correlate well with the changes in ecdysteroid titer during larval development (Niwa and Niwa 2016a,b). In addition, all known Halloween genes, except for *shd*, display high tissue specificity, as they are predominantly expressed in the PG (Niwa and Niwa 2014; Christesen *et al.* 2016; Ou *et al.* 2016; Nakaoka *et al.* 2017). Such temporally dynamic and spatially restricted expression profiles of the Halloween genes imply a tight transcriptional control network. To date, several transcription factors (TFs) have been implicated in the PG-specific regulation of the Halloween genes, including βFTZ-F1 (Parvy *et al.* 2005; Talamillo *et al.* 2013), Broad (Moeller *et al.* 2013), the CncC-dKeap1 complex (Deng and Kerppola 2013), DHR4 (Ou *et al.* 2011), Knirps (Danielsen *et al.* 2014), Molting defective (Neubueser *et al.* 2005; Ono *et al.* 2006; Danielsen *et al.* 2014), and Ventral veins lacking (Cheng *et al.* 2014; Danielsen *et al.* 2014). Although all these TFs are essential for the expression of ecdysteroidogenic genes in the PG, the tissue distribution of these TFs is not restricted to the PG, raising the question as to how the tissue specificity of ecdysone production is ensured.

In the fruit fly, the most recently identified ecdysteroidogenic TF is Ouija board (Ouib), which displays unique characteristics regarding spatial expression and *in vivo* function (Komura-Kawa *et al.* 2015). The *ouib* gene encodes a DNA-binding protein with five C_2_H_2_-type zinc finger motifs and an N-terminal protein domain known as zinc finger-associated domain (ZAD) (Chung *et al.* 2002). In contrast to other ecdysteroidogenic TFs, *ouib* is specifically expressed in the PG of *Drosophila melanogaster*. Null mutations of *ouib* resulted in developmentally arrested larvae and caused sharply reduced expression of a single Halloween gene, *spok*. Consistent with this finding, the regulatory region of *spookier* harbors a response element that appears to be specific to Ouib. Strikingly, the developmental arrest phenotype of *ouib* mutants was rescued by the overexpression of *spo*, a paralog of *spok* (*spok* overexpression had failed for technical reasons). These observations imply that the primary biological function of Ouib is to specifically regulate *spok* transcription during *Drosophila* development, which led us to propose that Ouib is the first identified invertebrate TF that is specialized for steroid hormone biosynthesis (Komura-Kawa *et al.* 2015; Niwa and Niwa 2016b).

The family of the ZAD-C_2_H_2_-type zinc finger genes underwent extensive duplication events and expansion during insect evolution (Chung *et al.* 2002). In the *D. melanogaster* genome, there are at least 98 ZAD-C_2_H_2_-type zinc finger genes (Chung *et al.* 2007). Besides Ouib, Molting defective (Mld) is another ZAD-C_2_H_2_-type zinc finger protein that is required for ecdysone biosynthesis (Neubueser *et al.* 2005; Ono *et al.* 2006; Danielsen *et al.* 2014). These findings raise the question as to whether additional ZAD-C_2_H_2_ zinc finger genes are involved in the control of ecdysteroidogenic gene expression in the PG and, if so, how these ZAD-C_2_H_2_ zinc finger family members functionally interact with each other.

Here, we describe a third ecdysteroidogenic ZAD-C_2_H_2_ zinc finger gene, designated *séance* (*séan*), which is crucial for ecdysone biosynthesis in the *D. melanogaster* PG. Remarkably, PG-specific expression of *nvd* rescues the lethality associated with a *séan* mutation. We demonstrate that Séan is of particular importance for the control of *nvd* expression through a specific element in the *nvd* promoter region. Moreover, both Séan and Ouib cooperatively act with Mld to positively regulate transcription of *nvd* and *spok*, respectively. Our genetic analysis also showed that we could rescue the larval arrest phenotype of *mld* mutants by the simultaneous overexpression of both *nvd* and *spok*. From an evolutionary perspective, Séan, Ouib, and Mld are found only in Drosophilidae species. Our study raises the intriguing possibility that the three ZAD-C_2_H_2_ zinc finger proteins Séan, Ouib, and Mld have specifically evolved to regulate the transcriptional activity of just two Halloween genes, *nvd* and *spok*, in *D. melanogaster*.

## Materials and Methods

### *Drosophila* strains

*Drosophila melanogaster* flies were reared on standard agar-cornmeal medium at 25° under a 12:12 hr light/dark cycle. *w^1118^* served as a control strain. *y^1^ v^1^ nos-phiC31*; *attP40*, *v^1^* and *y^2^ cho^2^ v^1^*; *attP40{nos-Cas9}/CyO* (Kondo and Ueda 2013) were obtained from the National Institute of Genetics, Japan. The Cas9-expressing line *y^[1]^M{vas-Cas9}ZH-2Aw^[1118]^/FM7c* (stock number #51323), a deficiency (*Df*) strain *w^1118^*; *Df(3R)BSC197/TM6B Tb* that lacks a genomic region that includes the *séan* locus (#9623) (Cook *et al.* 2012), and upstream activating sequence (*UAS)-CG8145(séan)-IR^TRiP.GL00720^* (#43551) (Perkins *et al.* 2015) were all obtained from the Bloomington *Drosophila* Stock Center. We received *UAS-CG8145(séan)-IR* (#35840 and #100854), *UAS-CG11762(ouib)-IR* (#108919), *UAS-CG8159-IR* (#35841), *UAS-CG9793(ranshi)-IR* (#197393), and *UAS-CG9797(M1BP)-IR* (#110498) from the Vienna *Drosophila* RNAi Center (VDRC). The *UAS-nvd-IR-1b* strain was previously described (Yoshiyama *et al.* 2006). *phm–GAL4#22* (McBrayer *et al.* 2007), *w*; *UAS-dicer2*; *phm-GAL4#22/TM6 Ubi-GFP*, *mld^47^* (Neubueser *et al.* 2005), and *mld^4425^* (Ono *et al.* 2006) were kind gifts from Michael B. O’Connor (University of Minnesota). *phm-GAL4* and *Feb36-GAL4* (Siegmund and Korge 2001; Andrews *et al.* 2002) were used as the strains to drive forced gene expression in the PG. *UAS-spo-HA* and *UAS-nvd-Bm[WT]-HA* were previously described (Namiki *et al.* 2005; Yoshiyama-Yanagawa *et al.* 2011).

### Generation of the *CG8145/séance* alleles

**Figure 1:**
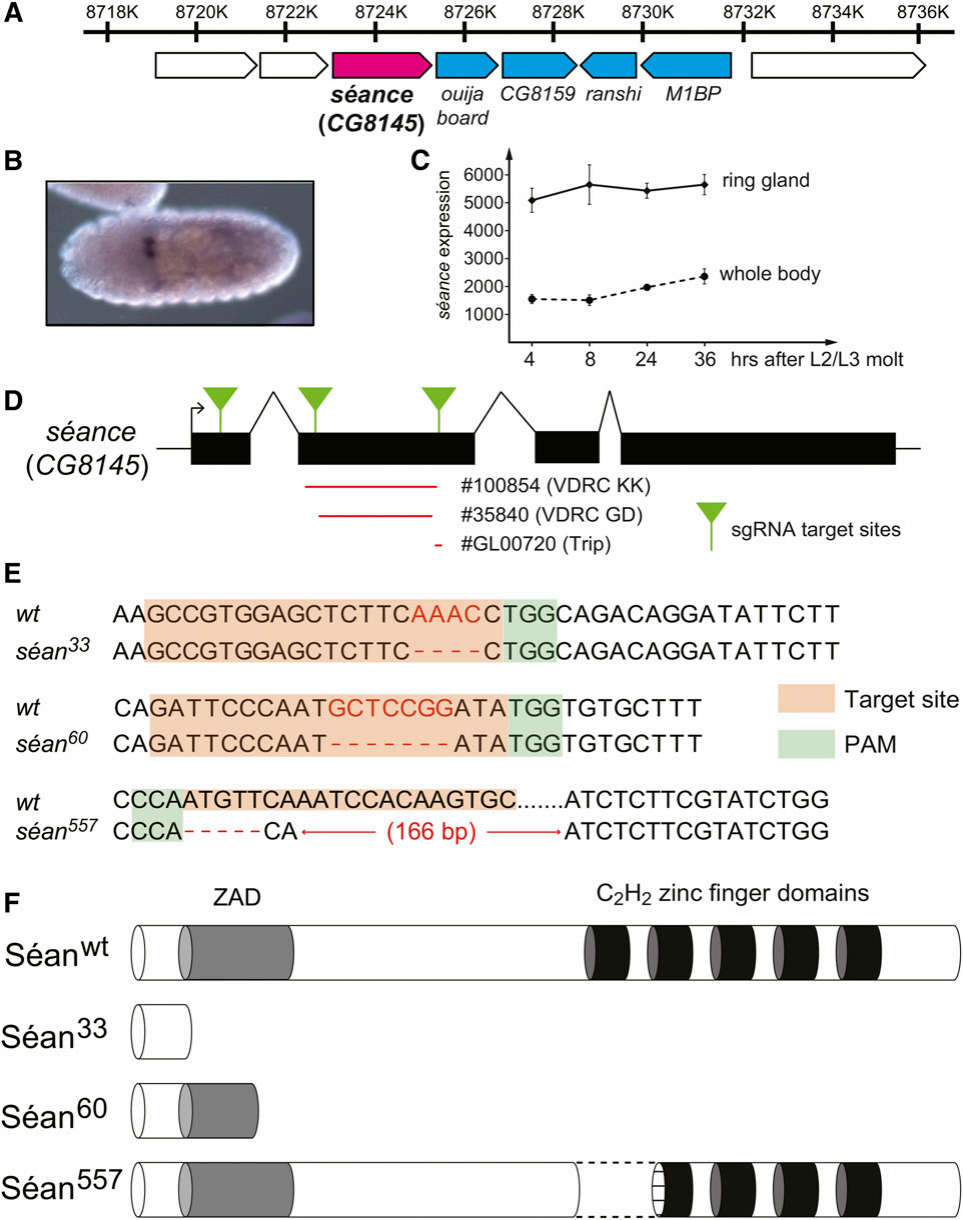
Generation of *séance (CG8145)* mutant alleles by the CRISPR/Cas9 system. (A) The genomic structure of *séance* and surrounding genes. The data are derived from the FlyBase GBrowse website (http://flybase.org/cgi-bin/gbrowse2/dmel/?Search=1;name=FBgn0037617). Numbers indicate the nucleotide positions at the 85A9 cytological location of the chromosome 3R scaffold. Boxed arrows represent gene spans and their directions. *séan* is shown in magenta. Four other ZAD-zinc finger protein genes are shown in cyan. (B) RNA *in situ* hybridization of *Drosophila* embryo with a *séan*-probe. (C) *Séan* expression profile based on four time points during the L3 stage, data retrieved from our previous study (Ou *et al.* 2016). (D) A schematic representation of the *séan* gene showing the sgRNA target sites. Exons are shown as black boxes, the transcription initiation site as an arrow, and sgRNA target sites as green triangles. (E) Sequences of sgRNA target sites and deleted regions of three isolated *séan* alleles: *séan^33^*, *séan^60^*, and sean^557^. The 20-bp target sequence corresponding to each target site is indicated in orange, the neighboring 5′-NGG (or 5′-CCN on the other strand) PAM in green, and the cleavage site of Cas9 is shown as red characters. Deleted regions are indicated by hyphens. (F) Predicted protein structures of *séan* alleles. Séan^33^ and Séan^60^ are composed of 33 and 60 amino acids, respectively. Séan^557^ is four amino acids longer than the wild-type protein, but lacks the first zinc finger domain entirely and part of the second zinc finger domain with an in-frame inappropriate amino acid stretch (dotted line). Also, see Figure S2 in File S1. CRISPR, clustered regularly interspaced short palindromic repeats; L3, third instar; PAM, protospacer adjacent motif; sgRNA, single-guide RNA; VDRC, Vienna *Drosophila* RNAi Center; wt, wild-type; ZAD, zinc finger-associated domain.

We generated *séan* alleles via a clustered regularly interspaced short palindromic repeats (CRISPR)/Cas9 system using the pBFv-U6.2 vector (Kondo and Ueda 2013) provided by the National Institute of Genetics, Japan. We selected three independent target sites (Figure 1D). To minimize off-target effects of the CRISPR/Cas9 system, we designed the single-guide RNA (sgRNA) sequence so that no match existed for any stretch of 15 bases on the third chromosome, using the CRISPR Optimal Target Finder at http://tools.flycrispr.molbio.wisc.edu/targetFinder/ (Gratz *et al.* 2014). Sense and antisense oligonucleotides corresponding to sgRNA target sequences are listed in Supplemental Material, Table S1 in File S2. We inserted annealed 5′-phosphorylated oligonucleotides into *Bbs*I-digested pBFv-U6.2 and *pU63-BbsI-chiRNA* vectors (Addgene). We injected vectors into the embryos of the *y^1^ v^1^ nos-phiC31*; *attP40* or *y^1^ M{vas-Cas9}ZH-2A*, *w^[1118]^/FM7c* strains, and performed Cas9-based gene targeting as previously described (Kondo and Ueda 2013). Genetic crosses and detection of indel (insertion/deletion) mutations at the *séan* locus were either conducted with T7 endonuclease (New England Biolabs, Beverly, MA) as previously described (Kondo and Ueda 2013; Komura-Kawa *et al.* 2015) or by PCR screening of F1 males after they had produced sufficient offspring (for primers see Table S1 in File S2). DNA fragments including the Cas9 target site were amplified by PCR with the extracted genome DNA from each strain, KOD FX Neo (TOYOBO, Osaka, Japan), and the primers (Table S1 in File S2) . Eventually, we isolated one strain for each target site for further analyses. These strains were renamed *séan^33^*, *séan^60^*, and *séan^557^*, all of which caused frameshift mutations within the *séan* ORFs (Figure 1, E and F and Figure S1 in File S1).

### Analyzing developmental progression of *séan* mutants

We crossed *séan^33^/TM3 Act-GFP* flies, *séan^60^/TM3 Act-GFP* flies, and *w^1118^* flies with each other. Eggs were laid on grape plates with yeast pastes at 25° for 8 hr. Thirty-six hours after egg laying (AEL), 100 hatched GFP-negative (*séan^33^/+*, *séan^60^/+*, and *séan^33^/séan^60^*) first-instar (L1) larvae were transferred to vials with a standard cornmeal diet (25 larvae per vial). Every 24 hr, larval stages were scored by tracheal morphology as previously described (Niwa *et al.* 2010).

### Quantification of 20E

For quantification with mass spectrometry, we collected L1 (36 hr AEL) for each genotype and determined the wet weight of each sample, after which samples were frozen in liquid nitrogen and stored at −80° until measurement. Extraction of steroids from whole larval bodies, HPLC fractionation, and mass-spectrometric analyses were previously described (Igarashi *et al.* 2011; Hikiba *et al.* 2013). In this study, the quantification range was 0.49–31.25 ng/ml, with a detection limit of 3.68 pg of 20E/mg (wet weight).

For quantification via enzyme-linked immunosorbent assay (ELISA), we harvested *séan^557^* embryos that were laid on grape juice plates within a 6-hr window and transferred them to food plates (standard cornmeal media) at 25°. Control (#51323; Bloomington) and *séan^557^* second-instar (L2) larvae were collected 12–18 hr after the L1/L2 molt. For PG-specific *séan*-RNAi (RNA interference), control (*phm>w^1118^*) and *phm*>*séan*-RNAi (#100854; VDRC) were staged at the L2/L3 (third instar) molt and collected 40–44 hr after the molt. Samples were then processed as previously described (Ou *et al.* 2011).

### RNA *in situ* hybridization

To generate the *séan* RNA probe, we constructed a pBluescriptII SK(−) (Promega, Madison, WI) plasmid containing the *séan* coding sequence (CDS), designated here as *séan*-pBluescript. Using the ReverTra Ace qPCR RT Kit (TOYOBO), we synthesized cDNAs from total RNA isolated from *w^1118^* larval ring glands. A DNA fragment representing the *séan* CDS was amplified by PCR from the cDNAs, and ligated to *Sma*I-digested pBluescriptII SK(−), yielding *séan*-pBluescript. Digoxigenin (DIG)-labeled antisense RNA probes were synthesized using DIG RNA-labeling mix (Roche Diagnostics, Basel, Switzerland) with T3 and T7 RNA polymerases (Thermo Fisher Scientific, Waltham, MA). Fixation, hybridization, and detection were performed as described previously (Lehmann and Tautz 1994; Niwa *et al.* 2004).

### RNA sequencing (RNA-Seq)

We carefully staged control (*phm>w^1118^*) and *phm*>*séan*-RNAi (VDRC #100854) larvae at the L2/L3 molt, and we dissected ring glands at 44 ± 0.5 hr after the molt. These samples were then transferred to ice-cold TRIzol reagent (Thermo Fisher Scientific). For each sample, the lysates of 20 ring glands were vortexed at room temperature for 5 sec, briefly spun down, and stored at −80° until use. Total RNA was isolated by NucleoSpin RNA (Macherey-Nagel, Düren Germany), quantified by RiboGreen Quanti Kit (Thermo Fisher Scientific), and RNA integrity was analyzed by Agilent Bioanalyzer Pico chips. We used 100 ng of total RNA per sample as input for cDNA library synthesis. Each genotype was analyzed by two independent biological replicates for analysis. The Encore Complete RNA-Seq library systems (NuGEN Technologies, San Carlos, CA) were used to produce the cDNA libraries for next-generation sequencing, following the manufacturer’s instructions. The cDNA libraries resulting from the Encore RNA-Seq systems were pooled together in equal concentrations for sequencing at McGill University and the Génome Québec Innovation Centre (Montréal, Canada). We normalized raw data with ArrayStar 4.0 (DNASTAR), and data were analyzed by ArrayStar 4.0, Access (Microsoft, Redmond, WA), and the Database for Annotation, Visualization and Integrated Discovery (DAVID) (Huang *et al.* 2009).

### Quantitative real-time PCR (qPCR)

RNA samples of wild-type adult ovaries, *séan^30^/sean^60^* L1 larvae, and Schneider 2 (S2) cells were isolated using the RNAiso Plus reagent (TaKaRa, Shiga, Japan). Genomic DNA digestion and cDNA synthesis were performed using the ReverTra Ace qPCR RT Kit (TOYOBO). qPCR was performed using the THUNDERBIRD SYBR qPCR Mix (TOYOBO) or Universal SYBR Select Master Mix (Applied Biosystems, Foster City, CA), with a Thermal Cycler Dice TP800 or TP870 system (TaKaRa). We used serial dilutions of a plasmid containing the ORF of each gene as a standard. The expression levels of the target genes were normalized to an endogenous control, *ribosomal protein 49* (*rp49*), (Foley *et al.* 1993) in the same sample. The primers for quantifying *séan* and *mld* are described in Table S1 in File S2. Primers used to amplify *nvd*, *sro*, *spok*, *phm*, *dib*, and *sad* were previously described (McBrayer *et al.* 2007; Niwa *et al.* 2010).

For all other samples, total RNA of whole larvae was isolated following a modified TRIzol protocol, where we substituted sodium acetate with lithium chloride for RNA precipitation. First, 10 ring glands or 10 CNS-ring gland complexes were dissected in ice-cold phosphate-buffered saline (PBS), rinsed twice with fresh PBS, transferred into TRIzol, and snap-frozen in liquid nitrogen. RNA of dissected tissues was extracted using RNeasy Mini (QIAGEN, Valencia, CA) or NucleoSpin RNA kits, following the manufacturers’ instructions. RNA samples (0.1–2 μg/reaction) were reverse-transcribed using an ABI High Capacity cDNA Synthesis kit (Thermo Fisher Scientific), and the synthesized cDNA was used for qPCR (QuantStudio 6 Flex Real-Time PCR System; Thermo Fisher Scientific) using KAPA SYBR Green PCR master mix (D-Mark) with 5 ng of cDNA template, with a primer concentration of 200 nM. Samples were calibrated to *rp49* based on the ∆∆C_t_ method. Primer sequences used for these samples are listed in Table S1 in File S2. The primer design ([T_m_] = 60 ± 1°) was based on the Roche online assay design center.

### Immunostaining

Immunostaining of ring glands was essentially performed as described previously (Imura *et al.* 2017). Dissected larval tissues were fixed in 4% paraformaldehyde in PBS + 0.3% Triton X-100 for 20 min at room temperature. Samples were then washed with PBS and incubated overnight at 4° with primary antibodies: guinea pig anti-Nvd (1:200) (Ohhara *et al.* 2015) and rabbit anti-Phantom (1:200; Phm) (Parvy *et al.* 2005). For this study, we used goat anti-guinea pig Alexa Fluor 488 and goat anti-rabbit Alexa Fluor 555 (Life Technologies, Carlsbad, CA) as fluorescent secondary antibodies. Secondary antibodies were diluted 1:200 and incubated for 1 hr at room temperature. Confocal images were captured using an LSM 700 laser scanning microscope (Zeiss [Carl Zeiss], Thornwood, NY).

### Sterol supplementation experiments

Twenty milligrams of dry yeast was mixed with 38 μl H_2_O and 2 μl ethanol, or supplemented with 2 μl of the following sterols dissolved in 100% ethanol: cholesterol (150 mg/ml; Wako, Osaka, Japan), 7DC (150 mg/ml; Sigma [Sigma Chemical], St. Louis, MO), ecdysone (10 mg/ml; Steraloids, Newport, RI), and 20E (50 mg/ml; Sigma). We crossed *séan^33^/TM3 Ser^1^ GMR2 Act-GFP* flies with *séan^60^/TM3 Ser^1^ GMR2 Act-GFP* flies. Eggs were laid on grape plates with yeast pastes at 25° for 12 hr. We distinguished *séan^33^/séan^60^* from other progenies by the presence or absence of GFP signal of the balancer chromosome. At 36 hr AEL, 50 hatched *séan^33^/séan^60^* L1 larvae were transferred to the yeast paste on grape plates and kept at 25°. Every 24 hr, developmental stages were scored by tracheal morphology.

### UAS vectors, overexpression of genes, GFP reporter constructs, and the generation of transgenic strains

The GAL4-UAS system (Brand and Perrimon 1993) was used to overexpress cDNAs in *D. melanogaster* both *in vivo* and in cultured S2 cells. For all vector constructions in this study, PCR was performed using KOD Plus Neo (TOYOBO).

To generate the *pUAST-séan*-cDNA construct, a *séan* cDNA clone (IP14660; *Drosophila* Genomics Resource Center, Indiana University) was first PCR-amplified, partially digested with *Eco*RI and *Xba*I, and cloned into the pUAST vector using the same enzymes. Transgenic flies carrying the *pUAST-séan*-cDNA construct were generated using conventional *P*-element transformation.

To generate a UAS vector to overexpress a cDNA encoding N-terminal V5 (GKPIPNPLLGLDST)-tagged Séan protein, we first made a pWALUM10-moe vector (Ni *et al.* 2011) with a V5-tag sequence. The oligonucleotides pWAL-V5-N-F and pWAL-V5-N-R (Table S1 in File S2) were annealed and then ligated to *Eco*RI-*Bgl*II-digested pWALIUM10-moe, leading to N-V5-pWALIUM10-moe. In parallel, we amplified the *séan* CDS by PCR with *séan*-pBluescript and specific primers [CG8145_N-V5_F and CG8145_N-V5_R (Table S1 in File S2)] to add *Nde*I and *Nhe*I sites to the 5′ and 3′ ends of the CDS, respectively. The PCR fragment was digested with *Nde*I and *Nhe*I, and then ligated into a *Nde*I-*Nhe*I-digested N-V5-pWALIUM10-moe.

To generate a UAS vector to overexpress *HA-mld*, which encodes N-terminal 3xHA-tagged Mld protein, we obtained a *Drosophila* Gateway vector (pTHW containing 3xHA sequences) from the Drosophila Genomics Resource Center (https://emb.carnegiescience.edu/drosophila-gateway-vector-collection#_References) (#1099). Specific primers, including a Gateway technology recognition sequence (CACC) at the N-terminus, were used for PCR (Table S1 in File S2). The *mld*-pUAST vector (Neubueser *et al.* 2005) (a gift from Stephan M. Cohen, University of Copenhagen, Denmark) was used as template DNA. We performed PCR using KOD Plus Neo (TOYOBO) and ligated the amplified *mld* CDS region into the pENTR TOPO vector (Thermo Fisher Scientific). This ENTRY vector and pTHW were mixed with LR clonase (Thermo Fisher Scientific), leading to *3xHA-mld*-pTHW.

To generate a UAS vector to produce N-terminal 3xTy1 (EVHTNQDPLD)-tagged CG8159 protein, we first made a pBluescript II SK(−) plasmid with a 3xTy1-tag sequence. The oligonucleotides Ty1_x3_For and Ty1_x3_Re (Table S1 in File S2) were annealed and then ligated to a *Sma*I-digested pBluescript II SK(−), leading to *3xTy1*-pBluescript. We amplified the 3xTy1 fragment with 5′ and 3′ extensions by PCR, using the *3xTy1*-pBluescript and the specific primers Ty1ForprimerpBlueGib and Ty1-Rev-primer (Table S1 in File S2). In parallel, we amplified the *CG8159* CDS by PCR with female whole-body-derived cDNA and the primers CG8159-Fwd-Gib and CG8159-Fwd-Gib (Table S1 in File S2). These two PCR fragments were ligated into a *Sma*I-digested pBluescript II SK (−) by the In Fusion Cloning Kit (TaKaRa). This ligated plasmid was digested by *Eco*RI and *Xba*I, and ligated it into *Eco*RI-*Xba*I-digested pWALIUM10-moe.

To generate a UAS vector to express a *D. melanogaster nvd* cDNA, we obtained a clone (#RE52861) from the Berkley *Drosophila* Genome Project (Rubin *et al.* 2000). The RE clone library was made from RNA extracted from *D. melanogaster* 0–22 hr mixed-stage isogenic *y*; *cn bw sp* strain embryos. Sequencing of the 1.7 kb *nvd* cDNA (RE52861) in the pFlc-1 vector revealed two point mutations resulting in amino acid substitutions without changing the reading frame: an AAA to AGA mutation in exon 1 resulting in a K17R substitution, and a CCT to ACT change in exon 2 resulting in a P159T substitution. The QuikChange Site-directed Mutagenesis kit (Stratagene, La Jolla, CA) was used to repair the two changes to the wild-type sequence. Primer sequences used to repair pFlc-1-RE52861 back to the wild-type sequence are described in Table S1 in File S2. The database sequence was confirmed by sequencing the background chromosome on which the mutations were induced as well as the wild-type chromosome. The repaired cDNA was subcloned from the pFlc-1 vector into the *Eag*I and *Kpn*I sites of pUAST.

### Genetic rescue experiments with *séan* and *nvd*

For rescue experiments of *séan* mutants by *nvd* overexpression, *w^1118^*; *séan^60^ phm-GAL4#22/TM6 Tb* and *w^1118^*; *séan^33^ UAS-nvd-Bm[WT]/TM6 Tb* were established by chromosomal recombination on the third chromosomes. The *w^1118^*; *séan^60^ phm-GAL4#22/TM6 Tb* flies were crossed with the *w^1118^*; *séan^33^ UAS-nvd-Bm[WT]]/TM6 Tb* flies. Eggs were laid on standard agar-cornmeal medium at 25° for 24 hr. *Tb^+^* L3 larvae, corresponding to *séan^60^ phm-GAL4#22/ séan^33^ UAS-nvd-Bm* animals, were collected and then survival larvae, pupae, and adults of the animals were scored.

For the rescue experiments of *mld* mutants by simultaneous expression of *nvd* and *spo*, *w^1118^*; *phm-GAL4#22 mld^47^/TM3* and *w^1118^*; *UAS-nvd(without tag) mld^4425^/TM3* flies were established by chromosomal recombination on the third chromosome. We also generated *w^1118^*; *UAS-spo-HA*; *phm-GAL4 mld^47^/TM3*. The flies of *w^1118^*; *phm-GAL4#22 mld^47^/TM3* were crossed with *w^1118^*; *mld^4425^/TM3*, and the flies of *w^1118^*; *UAS-spo-HA*; *phm-GAL4 mld47/TM3* were crossed with *w^1118^*; *UAS-nvd(without tag) mld^4425^/TM3*. The number of rescued adults of *mld^47^*/*mld^4425^* was scored.

For the rescue experiments of *séan^557^* mutants by *séan* overexpression, *séan^557^*, *phm22-GAL4/TM6B*, *Tb*, *Hu* flies were established by chromosomal recombination. *UAS-séan-cDNA (1M)*; *séan^557^/TM6B*, *Tb*, *Hu* flies were crossed to *séan^557^*, *phm22-GAL4/TM6B*, *Tb*, *Hu*. As a control, the flies of *séan^557^/TM6B*, *Tb*, *Hu* were crossed to *séan^557^*, *phm22-GAL4/TM6B*, *Tb*, *Hu*. *Tb^+^*, and progenies were scored for each cross after eclosion.

For the rescue experiments of *séan^557^* mutants by *nvd* overexpression, *séan^557^*, *phm22-GAL4/TM6B*, *Tb*, *Hu* and *séan^557^*, *UAS-nvd-cDNA(Bm) /TM6B*, *Tb*, *Hu* flies were established by chromosomal recombination. For homozygotes, the flies of *séan^557^*, *phm22-GAL4/TM6B*, *Tb*, *Hu* were crossed to *séan^557^*, *UAS-nvd-cDNA(Bm) /TM6B*, *Tb*, *Hu*. For *trans*-heterozygotes, the flies of *séan^557^*, *phm22-GAL4/TM6B*, *Tb*, *Hu* were crossed to s*éan^60^*, *UAS-nvd-cDNA(Bm) /TM6B*, *Tb*, *Hu*, and the flies of *séan^557^*, *UAS-nvd-cDNA(Bm)/TM6B*, *Tb*, *Hu* were crossed to *séan^33^*, *phm22-GAL4/TM6B*, *Tb*, *Hu*. As controls, the flies of *séan^557^/TM6B*, *Tb*, *Hu* were crossed to *séan^33^*, *phm22-GAL4/TM6B*, *Tb*, *Hu* and *séan^60^*, *UAS-nvd-cDNA(Bm)/TM6B*, *Tb*, *Hu*, respectively. *Tb^+^* progenies were scored for each cross after eclosion.

### Construction of *luciferase* reporter plasmids

We amplified a series of *nvd* upstream regions from *w^1118^* genomic DNA using primers (Table S1 in File S2) to add *Not*I and *Bgl*II sites to the 5′ and 3′ ends, respectively. These amplified *nvd* upstream regions were digested with *Not*I and *Bgl*II, and ligated into a *Not*I-*Bgl*II-digested pGL3-Basic vector luciferase reporter plasmid (Promega). We constructed reporter plasmids with mutated regions from the pGL3-Basic plasmid containing a wild-type upstream 301-bp region by inverse PCR with specific primers (Table S1 in File S2).

The +111 to +32 bp upstream region of *spok* was amplified from *w^1118^* genomic DNA by specific primers (Table S1 in File S2) to add *Sac*I and *Bgl*II sites to the 5′ and 3′ ends, respectively. DNA fragments of the +91 to +32 bp and the +71 to +32 bp upstream regions were prepared by annealing sense and antisense oligonucleotides (Table S1 in File S2) containing *Sac*I and *Bgl*II sites to the 5′ and 3′ ends, respectively. The amplified and annealed upstream regions of *spok* were digested with *Sac*I and *Bgl*II, and then ligated into a *Sac*I-*Bgl*II-digested pGL3-Basic plasmid. All other pGL3-Basic plasmids containing a series of *spok* promoter regions were previously described (Komura-Kawa *et al.* 2015).

### Transfection and luciferase reporter assays

S2 cells were seeded in 1 ml Schneider’s *Drosophila* Medium (Thermo Fisher Scientific) with 10% heat-inactivated fetal calf serum and penicillin–streptomycin solution (Wako) in a 24-well plate (TrueLine) 1 day before transfection. Transfection of S2 cells was performed with an *Actin5C-GAL4* construct (a gift from Yasushi Hiromi), UAS constructs, and a series of pGL3-Basic plasmids using the Effectene Transfection Reagent (QIAGEN), as previously described (Niwa *et al.* 2004). The Copia *Renilla* Control plasmid (#38093; Addgene) (Lum *et al.* 2003) was used as the reference. Construction of *V5-sean*-pWALIUM10-moe and *HA-mld*-pUAST is described above. The *UAS-FLAG-ouib* construct was described previously (Komura-Kawa *et al.* 2015), as was *UAS-GFP.RN3* (Niwa *et al.* 2002). The cells were incubated for 2 days after transfection. They were then processed using a Dual-Luciferase Reporter Assay System (Promega) following the manufacturer’s instructions, and were analyzed with Fluoroskan Ascent FL (Thermo Fisher Scientific).

### Data availability

Strains, DNA plasmids, and primers are available upon request. RNA-Seq data for Table 1, Table S2 in File S2, Table S3 in File S2, and Table S4 in File S2 are available at the Gene Expression Omnibus with the accession code GSE104340, associated with GSM2795618, GSM2795619, GSM2795620, and GSM2795621.

**Table 1:** Term enrichment for genes with differential expression in *séance*-RNAi ring gland samples

Term (*n*)	Number	Fold enrichment	*P*-value	Genes
Up (360)				
Ecdysone biosynthesis (8)	3	15.15	1.79E−10	***sad phm spok***
Oxidoreductase (275)	19	2.79	1.77E−06	*CG32557* ***Cyp18a1 sad phm*** *CG9512 CG7675 P5CDh1 Cyp4g1 CG5167 CG31937* ***spok*** *Cyp6a13 Trx-2 CG3719 Drat AOX3 Cyp6a9 Cyp317a1 v(2)k05816*
Glutathione transferase (39)	5	5.18	3.12E−05	*GstS1* ***GstE14/nobo*** *GstT4 GstD9 GstD10*
Cytochrome P450 (87)	8	3.72	5.19E−05	***Cyp18a1 sad phm*** *Cyp4g1* ***spok*** *Cyp6a13 Cyp6a9 Cyp317a1*
Nuclear receptor (21)	1	1.92	ns	***ftz-f1***
Down (248)				
Ecdysone biosynthesis (8)	1	7.33	1.83E−02	***nvd***
Oxidoreductase (275)	3	0.64	ns	*Cyp4e2 CG14946* ***nvd***
Glutathione transferase (39)	0	0	ns	*n/a*
Cytochrome P450 (87)	1	0.67	ns	*Cyp4e2*
Nuclear receptor (21)	4	11.17	8.07E−10	*knrl* ***Hr4 kni Eip75B***

Manually curated term enrichment analysis. For DAVID (the Database for Annotation, Visualization and Integrated Discovery)-based GO term analysis, see Tables S1 and S2 in File S2. Bold indicates genes with known roles in ecdysone homeostasis. (*n*) = presents how often the term is represented in the *Drosophila melanogaster* genome (*e.g.*, eight ecdysone biosynthetic genes). This analysis is based on 360 significantly upregulated and 248 significantly downregulated, as detected by RNA sequencing in *phm > séance*-RNAi ring gland samples *vs.* controls. *P*-values based on χ^2^ test. ns, not significant.

## Results

### The ZAD-C_2_H_2_ zinc finger gene *CG8145/séance* encodes a TF with essential roles in the PG

Given the role of the ZAD-C_2_H_2_ zinc finger protein Ouija board (Ouib) in controlling ecdysone biosynthesis in *D. melanogaster* (Komura-Kawa *et al.* 2015), we wondered whether other family members of the ZAD-C_2_H_2_ zinc finger family would also have roles in steroidal pathways. Interestingly, the *ouib* locus (85A9 on chromosomal arm 3R) is part of a cluster that comprises four additional ZAD-C_2_H_2_ zinc finger-encoding genes (*CG8145*, *CG8159*, *ranshi*, and *M1BP*). These genes are all paralogous to *ouib* and are presumably the result of tandem duplications (Figure 1A). When we conducted RNA *in situ* hybridization for these genes in *Drosophila* embryos, we noticed that *CG8145* had strong and specific expression in the ring gland (Figure 1B). A similar pattern has also been reported by the Berkeley *Drosophila* Genome Project Experiment IP14660 (Tomancak *et al.* 2007). Consistent with this, when we mined microarray data from a recently published study (Ou *et al.* 2016), we found that *CG8145* transcript levels were moderately enriched in the larval ring gland, but the expression level in the whole-body sample indicated that *CG8145* is expressed in other tissues as well (Figure 1C). In contrast, *CG8145* showed low expression levels in ovaries, a known source of ecdysteroids in adults (Figure S1 in File S1).

We then examined whether we could observe larval lethality when each of these ZAD-zinc finger protein genes was knocked down by PG-specific RNAi, for which we used the *phm22-GAL4* driver (hereafter *phm>*). In addition to *phm>ouib*-RNAi, we found that PG-specific RNAi against *M1BP* and *CG8145* resulted in larval lethality, whereas *CG8159*-RNAi and *ranshi*-RNAi did not. We omitted *M1BP* from further analysis in this study, as *M1BP* is known to encode a TF that regulates expression of non-TATA-type genes (Li and Gilmour 2013). On the other hand, *CG8145*, also known as “*numerous disordered muscles*” (short: *nom*) (Dobi *et al.* 2014), has no known roles in steroidogenesis, but given its transcript enrichment in the ring gland, we further investigated the function of this gene. Further testing with additional RNAi lines validated our initial findings, since three independent lines all gave the same results and two of the lines (#GL00720) did not overlap in their respective target sequences (Figure 1D). Given that we show here that *CG8145* is essential for regulating steroid hormone production, we renamed *CG8145* to *séance* (short: *séan*), in accordance with the tradition that genes encoding enzymes acting in the conversion of dietary sterols to ecdysteroids are collectively known as Halloween genes, and that it appears to work in conjunction with *ouija board*. A séance refers to a ritual that uses a ouija board to attempt communicating with the dead.

### *séance* is essential for larval development

To further assess the functional role of *séan*, we generated *séan* loss-of-function alleles by using a CRISPR/Cas9 approach (Kondo and Ueda 2013). We isolated three independent mutant alleles, *séan^33^*, *séan^60^*, and *séan^557^*, each of which was caused by a small deletion induced by different sgRNAs (Figure 1D). The *séan^33^* and *séan^60^* alleles lead to premature stop codons in the putative CDS of *séan* (Figure 1E), eliminating all five zinc finger domains in the C-terminal region of Séan (Figure 1F). In contrast, the *séan^557^* allele is caused by a deletion that removes part of exon 2 and its downstream intron (Figure 1E and Figure S2 in File S1). This deletion is predicted to cause an in-frame readthrough of the remaining intron 2 sequence, resulting in a stretch of inappropriate amino acids (Figure 1F, dotted line) that are incorporated into the protein, removing zinc finger 1 entirely and part of the second zinc finger (Figure 1F and Figure S2 in File S1).

**Figure 2:**
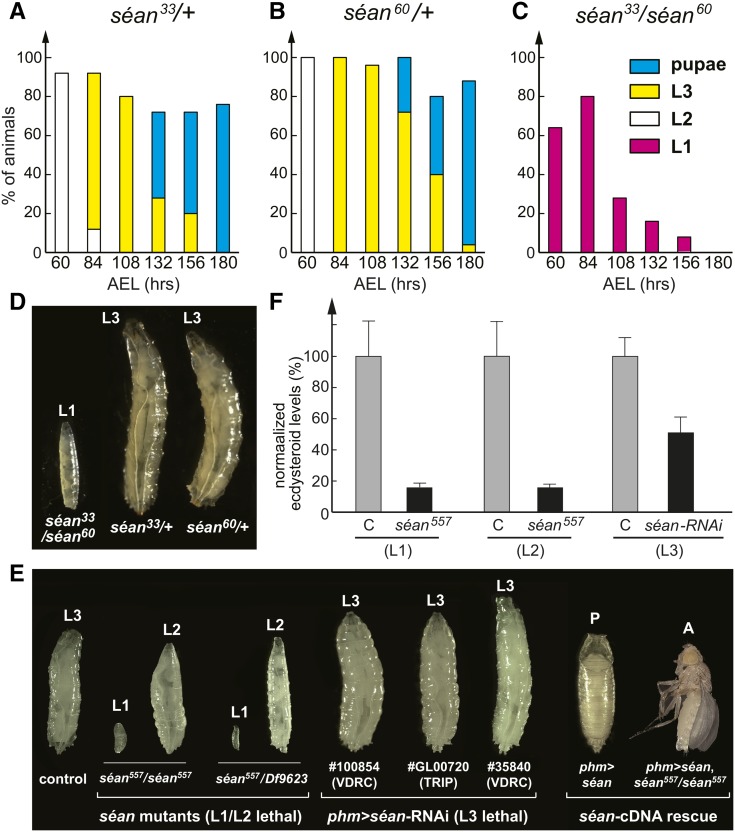
Larval lethality and developmental arrest phenotype of *séance* mutant larvae. (A–C) The survival rate and developmental progression of control (A and B) and *séan* mutant animals (C), each at *N* = 50. (D) Comparison of body size and developmental stage between control (right and middle) and *séan^33^/séan^60^* mutants (left) at 108 hr AEL. Control animals developed into L3 larvae and adults (not shown), whereas *séan* mutants showed arrested development as L1 larvae. (E) Phenotypic comparison of *séan^557^* mutants, *séan*-RNAi, and a rescue with *séan*-cDNA. *séan^557^* mutants arrest as L1 and L2, whereas PG-specific expression of *séan*-RNAi (*phm>séan*-RNAi, VDRC #100854) results in L3 lethality. Homozygous *séan^557^* mutants were rescued by PG-specific expression of *séan^557^* cDNA. (F) Whole-larvae ecdysteroid quantification. Control and *sean^557^* homozygous mutants were compared during L1 (6–18 hr after egg hatch) and L2 (12–18 hr after L1/L2 molt). PG-specific *séan*-RNAi (VDRC #100854) L3 was compared to that of control animals at 40–44 hr L3. At least three samples were tested per genotype in each data set, and each sample was tested in triplicate. Error bars represent SE. Percentages were normalized to control levels of each data set. For L1, *N* = 300 for each genotype; for L2, *N* = 90 for each genotype; and for L3, *N* = 24 for each genotype. AEL, after egg laying; L1, first instar; L2, second instar; L3, third instar; PG, prothoracic gland; RNAi, RNA interference; TRIP, Transgenic RNAi Project; VDRC, Vienna *Drosophila* RNAi Center.

*séan^33^/séan^60^* animals exhibited an early larval (L1) arrest phenotype, with no animals developing into final instar larvae, pupae, or adults (Figure 2, A–D). When we combined the *séan^33^* or *séan^60^* allele with a *Df* line that uncovers the *séan* locus (*séan^33^/Df* or *séan^60^/Df*), we obtained comparable results, suggesting that both s*éan^33^* and *séan^60^* are null alleles. Eventually, all *séan^33^/séan^60^* transheterozygous animals died by 180 hr AEL retaining L1-like morphology (Figure 2, C and D). In contrast, the majority of control *séan^33^/+* or *séan^60^/+* heterozygous animals became pupae by this time (Figure 2, A and B).

Larvae carrying two copies of the *séan^557^* allele died as L1 and L2 larvae, as did *séan^575^/Df* hemizygotes (Figure 2E). Although *séan^33^/séan^60^* transheterozygotes tended to produce nonmolting oversized L1 larvae (Figure 2C), *séan^557^* homozygotes, as well as *séan^575^/Df* hemizygotes, produced oversized L2 larvae, with the latter to a lesser degree. PG-specific expression of the three *phm>séan-RNAi* lines caused developmental arrest in the L3 stage, which also produced larvae that were larger than controls (Figure 2E).

Apart from the overgrowth phenotype, none of the allelic combinations showed any apparent developmental or morphological defects during embryonic or larval stages, suggesting that these animals lacked a signal to progress with development. Based on its similarity and vicinity to *ouib*, we hypothesized that *séan* had a similarly important role and that the developmental arrest in *séan* loss-of-function animals was caused by a lack or reduction of systemically acting steroid hormones.

### The *séance* loss-of-function phenotype is caused by ecdysteroid deficiency

We next examined whether the larval arrest and lethality phenotype of *séan* loss-of-function animals was caused by a failure to produce sufficient ecdysteroids. We used two experimental approaches to determine ecdysteroid concentrations. First, we examined ecdysteroid titers in L1 larvae (36 hr AEL) of controls and *séan^33^/séan^60^* transheterozygotes by mass-spectrometric analysis. In the control larvae, we detected 9.54 ± 0.96 pg of 20E/mg of wet weight (mean ± SEM, *N* = 4). In contrast, ecdysteroid titers in *séan^33^/séan^60^* animals (*N* = 5) were below the detectable limit, suggesting that loss of *séan* function severely impaired ecdysone biosynthesis during larval stages. Second, to confirm these findings, we used an ELISA approach to quantify ecdysteroid concentrations in *s*é*an^557^* and *phm>séan*-RNAi animals. This method also showed significantly lower ecdysteroid levels in either of these genotypes when compared to those of controls (Figure 2F).

**Figure 3:**
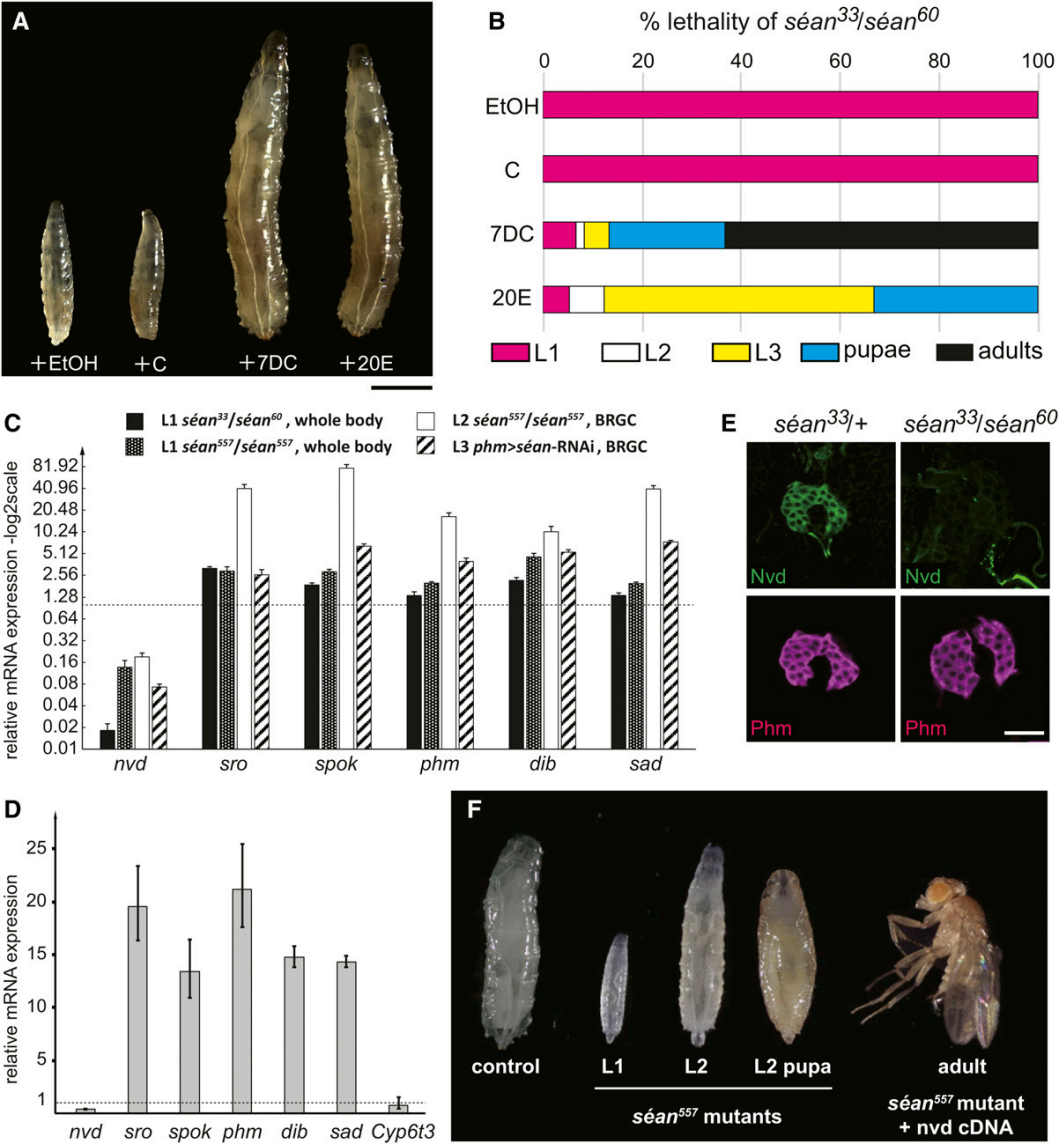
Expression analysis of Halloween genes and feeding rescue experiment in *séan* mutant larvae. (A) Rescue studies for *séan^33^/séan^60^* larvae. Mutant animals fed 20-hydroxyecdysone (20E) and 7-dehydrocholesterol (7DC) developed into third-instar (L3) larvae, whereas animals reared on cholesterol- and ethanol-containing food (vehicle control) remained first-instar (L1) larvae. Bar, 1 mm. (B) The survival rate and developmental progression of *séan^33^/séan^60^* mutant animals by oral administration of sterols and ecdysteroids (each *N* = 60). (C) Relative expression levels [quantitative PCR (qPCR)] of Halloween genes compared to those of controls (dotted line = 1) and various backgrounds of *séan* mutant or *séan*-RNAi (RNA interference) (*phm>séan*-RNAi, Vienna *Drosophila* RNAi Center #100854). BRGC, the brain-ring gland complex. Error bars indicate SEM. * *P* < 0.05 and ** *P* < 0.01 with Student’s *t*-test (black columns) and 95% C.I.s (all other columns). (D) Relative expression levels (qPCR) of Halloween genes in *nvd*-depleted prothoracic gland (PG). Expression levels were normalized to controls (dotted line = 1). *nvd*-RNAi was driven by the *Feb36-GAL4* driver, and the BRGCs were dissected from late L3 larvae, collected at 44 hr after the second-instar (L2)/L3 molt. Error bars indicate 95% C.I.s. (E) Immunostaining of the PG cells from control and *séan* mutant L1 larvae at 36 hr after egg laying with antibodies against Phm (magenta) and Nvd (green). Bar, 25 μm. (F) Expression of *nvd*-cDNA in a *sean^557^* mutant background rescued L1/L2 lethality to adulthood. L2 pupae form in rare cases using this genetic background; these animals attempt pupariation directly from L2 larvae.

Although the PG-specific disruption of ∼1200 genes via RNAi caused larval arrest phenotypes (Danielsen *et al.* 2016), it appears that only a fraction of these are directly involved in ecdysone production and its regulation. Consequently, only a few of these RNAi lines can be rescued all the way to adulthood by feeding ecdysone or 20E, a strategy that works very well for Halloween gene loss-of-function lines (Ou *et al.* 2016). When we tested whether *séan* mutants could be rescued with dietary ecdysone supplementation, we found that both *séan^33^/séan^60^* and *séan^557^*/*séan^557^* animals were partially rescued by 20E feeding. Specifically, *séan^33^/séan^60^* transheterozygotes and *séan^557^* homozygotes now reached pupal stages, some of the latter even reaching adulthood (Figure 3, A and B and Figure S3 in File S1). Taken together, these results indicated that loss of *séan* function caused ecdysteroid deficiency.

### *phm>séance*-RNAi ring glands exhibit reduction of *neverland* expression, but not other ecdysone biosynthetic genes

To identify downstream targets of Séan, we carried out RNA-Seq on samples from hand-dissected ring glands isolated from *phm>séan*-RNAi and control L3 larvae. We collected carefully staged larvae (44 ± 0.5 hr after L2/L3 molt) and dissected ring gland RNAs for transcriptome analysis. We collected 20 ring glands per sample to average out inherent differences in developmental timing, thus accounting for individuals that deviated from the population mean. Using a threefold cutoff for differentially expressed genes, we identified 360 up- and 248 downregulated genes. When we subjected these gene cohorts to GO term analysis via DAVID (Huang *et al.* 2009), the top term in the upregulated group was for genes involved in oxidation–reduction processes that included three genes involved in ecdysone biosynthesis: *phantom* (*phm*), *shadow* (*sad*), and *spookier* (*spok*) (Table S2 in File S2). Consistent with this, other terms including “steroid biosynthesis,” “cholesterol homeostasis,” “ecdysone biosynthesis,” and “glutathione metabolism” were also enriched among the upregulated gene set, all of which harbored genes with established links to ecdysone production (Enya *et al.* 2014; Danielsen *et al.* 2016). In contrast, the TF term was found to be enriched in the downregulated gene set and also harbored genes with known links to ecdysteroid regulation, such as *broad*, *knirps*, and *E75* (Table S3 in File S2). Based on these findings, we conducted a manual term enrichment analysis based on either gene function (*e.g.*, the eight known Halloween genes) or protein family (*e.g.*, P450 and nuclear receptor genes) (Table 1) to complement, refine, and correct errors intrinsic to the DAVID analysis. By comparing the same terms for both up- and downregulated genes, we found that not only genes associated with ecdysone biosynthetic processes were upregulated, but that a known inhibitor of ecdysone production, HR4, was downregulated. Notably, however, a single steroid biosynthetic gene, *neverland* (*nvd*), was nearly 10-fold downregulated (fold changes for genes listed in Table 1 are shown in Table S4 in File S2), suggesting that ecdysone production was impaired, and that the upregulation of the ecdysone biosynthetic pathway was an attempt by PG cells to compensate for overall low ecdysone production due to reduced *nvd* expression.

To validate these findings, we performed qPCR analysis to examine expression levels of six Halloween genes in both *séan* mutants and PG-specific *séan*-RNAi larvae. Consistent with the RNA-Seq data, the only gene with reduced expression was *nvd*, which was, dependent on the sample, 7- to 50-fold downregulated (Figure 3C). In contrast, *sro*, *spok*, *phm*, *dib*, and *sad* all showed moderate to substantial upregulation (∼2- to 70-fold) in *séan* loss-of-function animals. Curiously, *nvd*-RNAi animals also showed substantial upregulation of the same Halloween genes (Figure 3D). These results suggest that upregulation of *sro*, *spok*, *phm*, *dib*, and *sad* transcripts are not directly affected by the loss of *séan* function, but rather by impairment of cholesterol and/or 7DC metabolism in the PG.

Immunohistological analysis using anti-Nvd antibodies demonstrated that the Nvd protein level was also markedly reduced in *séan^33^/séan^60^* larvae compared to that in control animals, but not that of Phm, another ecdysone biosynthetic enzyme in the PG (Figure 3E). Therefore, we hypothesized that the resulting larval arrest phenotype was caused by reduced expression of *nvd* and not linked to the increase in the expression of other Halloween genes.

### *séance* is required for the conversion of cholesterol to 7DC

Nvd plays a crucial role in the first step of the ecdysone biosynthesis pathway, namely the conversion of dietary cholesterol to 7DC (Yoshiyama *et al.* 2006; Yoshiyama-Yanagawa *et al.* 2011). We have previously demonstrated that the larval arrest phenotype of loss-of-*nvd*-function animals is rescued by feeding 7DC but not cholesterol (Yoshiyama *et al.* 2006). If *séan* is required for the regulation of *nvd* during *Drosophila* development, we would expect that the larval arrest phenotype of *séan* should also be rescued by feeding of 7DC. Indeed, when *séan^33^/séan^60^* transheterozygotes were fed yeast paste supplemented with 7DC, they were rescued to pupal and adult stages, whereas cholesterol was unable to do so (Figure 3, A and B). Similarly, when we repeated the experiment with *séan^557^* homozygotes, only 7DC could rescue these animals to adulthood efficiently, whereas cholesterol, ecdysone, and 20E all failed to do so (Figure S3 in File S1). We noted that the higher rescuing activity of 7DC as compared to 20E was also observed in previous studies using loss-of-function animals for *nvd* and other genes required for cholesterol trafficking and metabolism (Table S5 in File S2) (Huang *et al.* 2005; Yoshiyama *et al.* 2006; Enya *et al.* 2014). 7DC can be utilized as a dietary precursor to generate a normal temporal fluctuation of ecdysteroids by a series of the biosynthesis enzymes downstream of Nvd. These results suggest that loss of *séan* function specifically impairs the catalytic conversion of cholesterol to 7DC. These results also demonstrate that the moderate increase in the expressions levels of *nobo*, *sro*, *spok*, *dib*, and *sad* does not contribute significantly to the *séan* mutant phenotype.

### The *séance* mutant phenotype is caused by the loss of *neverland* expression in the PG

We next examined whether the *séan* mutant phenotype was rescued by forced expression of *nvd* using the GAL4-UAS gene expression system. We utilized the UAS-*nvd-Bm* strain to overexpress the silkworm *Bombyx mori* ortholog of the *nvd* gene, as we have previously reported that *nvd-Bm* can rescue the larval arrest phenotype of *nvd*-RNAi in *D. melanogaster* flies (Yoshiyama-Yanagawa *et al.* 2011). We found that expression of *nvd-Bm* in the PG recovered the larval arrest phenotype of *séan^33^/séan^60^* transheterozygotes, which caused as much as 82.6% of animals to reach the adult stage (Figure 3F and Table 2). These results strongly suggest that the developmental arrest phenotype of *séan* mutants is caused solely by the loss of *nvd* expression in the PG. Therefore, in conjunction with our previous identification of Ouib (Komura-Kawa *et al.* 2015), our data support the idea that Séan and Ouib are functionally specialized to regulate the expression of two distinct Halloween genes, *nvd* and *spok*, respectively, during development.

**Table 2:** Rescue of *séance* mutants with PG-specific expression of a *neverland* cDNA

Genotype	Phenotype
*séan^33^*/*séan^60^*	L1 lethality
*phm-Gal4*	Survival to adulthood
*UAS-nvd*-cDNA	Survival to adulthood
*phm-Gal4*; *séan^33^*/*séan^60^*	L1 lethality
*UAS-nvd*-cDNA; *séan^33^*/*séan^60^*	L1 lethality
*phm-Gal4*, *séan^33^*/ *séan^60^*, *UAS-nvd*-cDNA	Survival to adulthood
*séan^557^*/*séan^557^*	L1 and L2 lethality
*phm-Gal4*, *séan^557^*/*séan^557^*, *UAS-nvd*-cDNA	Survival to adulthood
*séan^60^*/*séan^557^*	L1 and L2 lethality
*phm-Gal4*, *séan^60^*/*séan^557^*, *UAS-nvd*-cDNA	Survival to adulthood
*séan^33^*/*séan^557^*	L1 and L2 lethality
*phm-Gal4*, *séan^33^*/*séan^557^*, *UAS-nvd*-cDNA	Survival to adulthood

L1, first instar; L2, second instar.

### The *neverland* promoter is activated by cotransfection of *séance* and *molting defective* in S2 cells

We have previously identified the Ouib response element in a ∼170-bp genomic region upstream of the *spok* CDS. Moreover, in *D. melanogaster* S2 cells, the presence of Ouib is sufficient to drive a reporter *luciferase* (*luc*) gene fused with the 170 bp *spok* promoter (Komura-Kawa *et al.* 2015). Analogous to our previous findings, we focused on the identification of the *cis*-regulatory element(s) responsible for the Séan-mediated control of *nvd* expression using the *luc* assay system in S2 cells. We generated DNA constructs carrying the upstream region of *nvd* fused with a *luc* gene cassette, and then transfected S2 cells using these DNA constructs with or without a plasmid for overexpressing *V5-tagged-séan* (*V5-séan*). However, we failed to detect Séan-induced *luc* expression when we introduced the *V5-séan* plasmid alone into S2 cells, even with a 5-kb genomic region upstream of the translation initiation site of *nvd* (data not shown).

**Figure 4:**
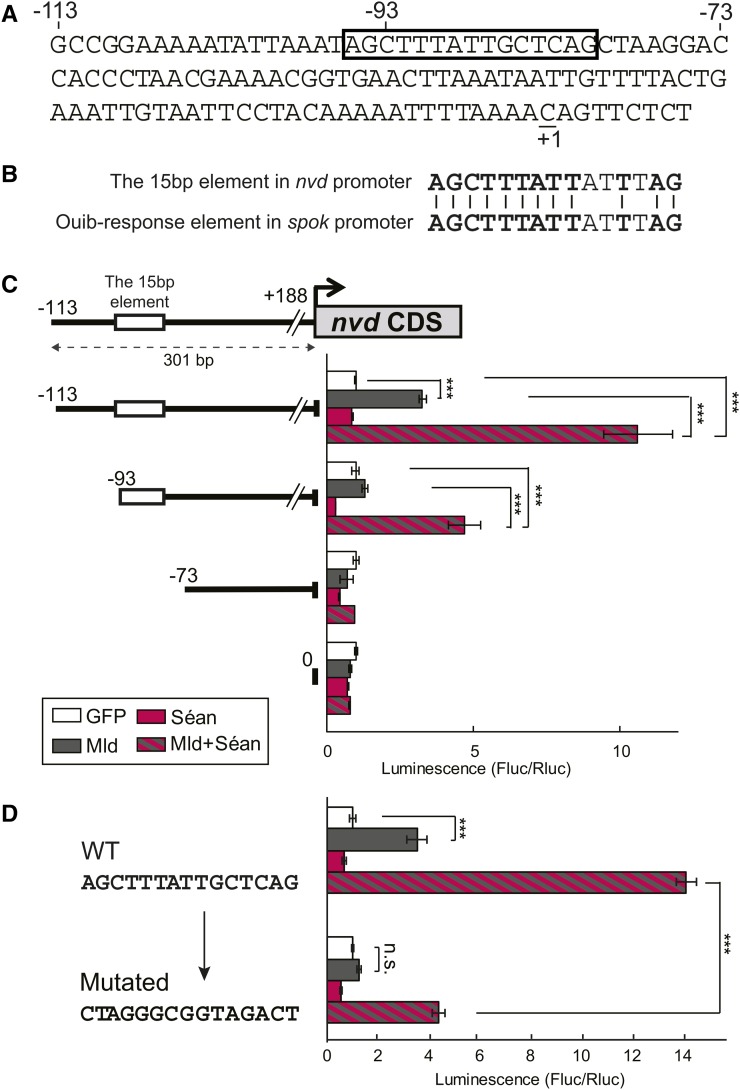
Transcriptional activity of Séan and Mld for the upstream element of *nvd*. (A) Schematic representation of the location of the element (−113 to −93) in the *D. melanogaster nvd* promoter region responsible for Séan-dependent transcriptional activation. Numbers indicate the distance from the transcription start site (+1, underlined) of *nvd*, which is based on FlyBase data (http://flybase.org/reports/FBgn0259697.html). The box indicates the 15-bp Séan-Mld response element. (B) The 15-bp element marked by the box in A exhibits a striking similarity to the Ouib response element in the *spok* promoter (15 bp). The bold letters and black lines indicate matching bases in the alignment between the element in the *nvd* promoter and the Ouib response element. (C) Luciferase reporter assay with plasmids containing the series of upstream elements of *nvd*. Numbers indicate the distance from the transcription start site of *nvd*. The white box indicates the Séan-Mld response element. The gray box represents the *nvd* CDS. Reporter activities of progressive deletion constructs are shown on the right (each at *N* = 3). The GFP expression plasmid was used as a negative control. (D) Luciferase reporter assay with plasmids containing the 9-bp transversion mutation in the −113 to −93 region of the 300-bp upstream element of *nvd* (each at *N* = 3). The GFP expression plasmid was used as a negative control. Error bars indicate SEM. *** *P* < 0.005 using Student’s *t*-test with Bonferroni correction. CDS, coding sequence; WT, wild type.

Although several possibilities exist that would explain this unsuccessful reconstruction, we reasoned that there might be a coregulator(s) acting in conjunction with Séan that is required to induce *nvd* expression in S2 cells. A candidate for such a coregulator is another ZAD-C_2_H_2_ zinc finger protein, Molting defective (Mld), since *mld* mutants exhibit *nvd* expression (Danielsen *et al.* 2014). Mld is, like Séan, a ZAD-C_2_H_2_ zinc finger protein (Neubueser *et al.* 2005; Ono *et al.* 2006; Danielsen *et al.* 2014). We found that transfection of *HA-tagged-mld* (*HA-mld*) plasmid fused with 301 bp upstream of the *nvd* CDS, including the 113 bp promoter region and 188 bp 5′ untranslated region, in S2 cells resulted in the induction of *luc* reporter activity (Figure 4, A–C). Moreover, coexpression of both *V5-séan* and *HA-mld* caused further drastic induction of the 301-bp *nvd* promoter-*luc* expression (Figure 4C). These results suggest that the first 301 bp upstream of the *nvd* CDS might contain one or more essential *cis*-regulatory elements that are required for Séan and Mld function.

### Identification of a Séance-molting defective-response element in the *neverland* promoter region

To narrow down the element(s) responsible for the Séan-Mld-dependent expression of *nvd*, we tested several constructs carrying the upstream region of *nvd* with a range of deletions within the 301-bp region. We first generated the deletion constructs in 20-bp increments from the 5′-terminus of the 301-bp region. Whereas the *luc* construct fused with the *nvd* promoter lacking the region from −113 to −94 bp was not activated by Mld alone, *luc* expression from this construct was still highly induced in the presence of both Mld and Séan (Figure 4C). In contrast, the region from −93 to −74 bp was crucial for the Séan-Mld-dependent *luc* reporter activity (Figure 4, B and C). Strikingly, this 20-bp region contains a DNA sequence 5′-AGCTTTATTGCTCAG-3′ that is nearly identical to the Ouib response element in the *spok* promoter region (5′-AGCTTTATTATTTAG-3′; underlines indicate the exact matches between the two sites) (Figure 4B) (Komura-Kawa *et al.* 2015). To clarify the importance of this 15-bp region for Séan-Mld-dependent control of gene expression, we introduced transversion mutations within the first 9 bp of this putative 15-bp recognition site. This mutated construct exhibited no *luc* reporter induction in the presence of Séan and Mld upon transfection into S2 cells (Figure 4D). These results suggest that the 15 bp of the *nvd* promoter region (from −95 to −81 bp) might serve as a Séan-Mld response element, while Mld also acts on the region from −113 to −94 bp.

We also examined the evolutionary conservation of *séan* as well as the Séan-Mld response elements in putative *nvd* promoter regions from other *Drosophila* species. Among 12 Drosophilidae species for which genome annotations have been reported (Clark *et al.* 2007), clear orthologs of *séan* are found in many, but not all, Drosophilidae species (Figure S4 in File S1). To examine the Séan-Mld response elements, DNA sequences around the *nvd* loci of *D. melanogaster*, *D. simulans*, *D. sechellia*, and *D. willistoni* were available. Using EMBOSS Matcher, an algorithm to identify local similarities between two sequences (McWilliam *et al.* 2013), we found that the Séan-Mld response element-like motifs were located in proximity (within 300 bp) to the *nvd* coding region in all of these four species (Figure S5 in File S1). In particular, the putative *nvd* promoter regions of *D. simulans* and *D. sechellia* contain the same 15-bp sequence motif. In conjunction with our previous analysis on the *spok* promoter (Komura-Kawa *et al.* 2015), these data suggest that both Ouib and Séan-Mld response elements are also evolutionarily conserved, at least in some *Drosophila* species.

### The *spookier* promoter is synergistically activated by Ouija board in the presence of molting defective in cultured S2 cells

**Figure 5:**
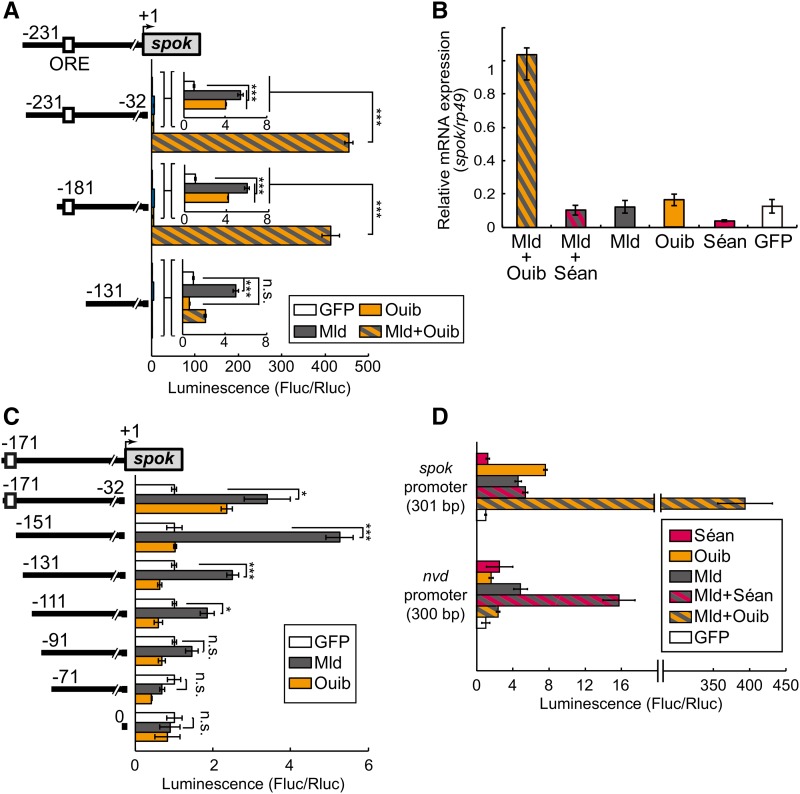
Transcriptional activity of Ouib and Mld for the upstream element of *spok*. (A) Luciferase reporter assay using the expression of *ouib* and *mld* along with plasmids containing the series of upstream elements of *spok*. Numbers indicate the distance from the translation, but not transcription, start site (+1) of *spok*, as a transcription start site for *spok* has not been defined. The numbering style of this study is exactly the same as that of Komura-Kawa *et al.* (2015). The white box indicates the Ouib response element (ORE). The gray box represents the *spok* coding region. The inset is an enlarged view of the transcriptional activity of Ouib, Mld, and GFP for the upstream element of *spok*. Reporter activities of progressive deletion constructs are shown on the right (each at *N* = 3). The GFP expression plasmid was used as a negative control. (B) Quantitative PCR analysis to measure expression levels of endogenous *spok* expression in S2 cells transfected with various expression constructs, each at *N* = 3. (C) Luciferase reporter assay (each at *N* = 3) with the *mld* expression plasmid and *luc* plasmids containing the series of upstream elements of *spok*. (D) Luciferase reporter assay (each at *N* = 3) with *séan*, *ouib*, and/or *mld* expression plasmids, and *luc* plasmids containing the upstream elements of *spok* (the +331 to +32-bp region; 301 bp) and *nvd* (the +300 to +1-bp region; 300 bp). The GFP expression plasmid was used as a negative control. Error bars indicate SEM. * *P* < 0.01 and *** *P* < 0.005 using Student’s *t*-test with Bonferroni correction. Fluc/Rluc, firefly luciferase/Renilla luciferase.

Unlike Séan, as we have previously reported (Komura-Kawa *et al.* 2015), Ouib alone had the ability to activate gene expression via the Ouib response element present in the *spok* promoter region (Figure 5A). On the other hand, the previous study has also reported that *mld* mutants also displayed decreased expression of not only *nvd* but also *spok* (Ono *et al.* 2006; Danielsen *et al.* 2014). Given that Séan and Mld cooperatively activate *nvd* promoter activity, we next examined if Ouib-mediated activation on gene expression was also synergistically enhanced in the presence of Mld. Strikingly, coexpression of both *FLAG-ouib* and *HA-mld* drastically increased *luc* induction under control of the 230- and 181-bp *spok* promoter regions, both of which contain the Ouib response element (Figure 5A). In contrast, the synergistic transactivation by Mld was not observed with the 131-bp *spok* promoter fragment lacking the Ouib response element (Figure 5A). Moreover, endogenous *spok* expression in S2 cells was drastically induced by coexpression of both *HA-mld* and *FLAG-ouib* (Figure 5B), whereas coexpression of *HA-mld* and *V5-séan* could not induce endogenous *nvd* expression (Figure S6 in File S1). These results indicate that Mld also works with Ouib to induce *spok* expression.

We also found that transfection of the *HA-tagged-mld* (*HA-mld*) plasmid alone in S2 cells induced *luc* reporter gene expression when we used the 170-bp upstream region of the *spok* CDS that completely lacked the Ouib response element (Figure 5C). We further generated the deletion constructs in 20-bp increments from the 5′ terminus of the 170-bp *spok* promoter region. We still observed Mld-mediated activation of *luc* reporter gene expression with the 110-bp region, but not the 90-bp region, of the *spok* promoter (Figure 5C). These results suggest that Mld acts on distinct regions from Ouib response elements in the *spok* promoter regions.

### There is no cross-reactivity between Séance and Ouija board

We next examined whether Séan and Ouib are specific to their respective recognition elements, or whether they can cross-react to activate either the *spok* or *nvd* promoters. Coexpression of *HA-mld* and *FLAG-ouib* did not induce *nvd* promoter-*luc* expression in cultured S2 cells (Figure 5D). In addition, coexpression of *HA-mld* and *V5-séan* did not induce *spok* promoter-*luc* expression in S2 cells (Figure 5D). We also observed that the expression of *3xTy1-tagged CG8159*, the paralogue of *séan* and *ouib* (Figure 1A), induced neither *nvd* promoter- nor *spok* promoter-*luc* expression with or without *HA-mld* (Figure S7 in File S1). These results suggest that the action of the paralogous ZAD-zinc finger TFs is highly specific to the *nvd* and *spok* promoters, respectively.

### The larval arrest phenotype of *molting defective* is rescued by forced expression of both *neverland* and *spookier*

Finally, we wondered whether the only essential targets of Mld are *nvd* and *spok*, or whether Mld also regulates other genes required for survival. To address this question, we expressed both *UAS-nvd* and *UAS-spo* in the PG cells of *mld* transheterozygotes using the PG-specific GAL4 driver (Table 3). It has previously been reported that *spo* overexpression can rescue the larval arrest phenotype of *spok* RNAi animals, confirming that *spo* and *spok* are functionally equivalent *in vivo* (Komura-Kawa *et al.* 2015), but that *spo* overexpression alone does not rescue the *mld* phenotype (Ono *et al.* 2006). Indeed, we found that the lethality of *mld* transheterozygotes was rescued by the overexpression of both *nvd* and *spo*. These results suggest that *nvd* and *spok* are the two major essential targets of Mld.

**Table 3:** Joint PG-specific expression of *spo* and *nvd* rescues *mld* mutants

Transgenes	F1 genotype
None	0 (358)
*UAS-spo and UAS-nvd*	35 (317)

The number of viable *mld^258^/mld^4425^* transheterozygous adults was scored. Values in parentheses indicate the number of viable control non-*mld* mutant progenies after genetic crosses described in the *Materials and Methods*.

## Discussion

**Figure 6:**
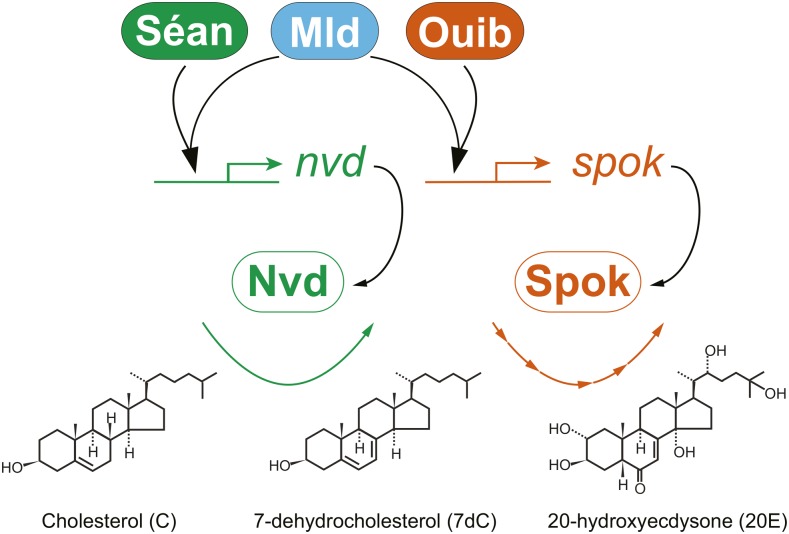
Model for transcriptional interaction between Séan, Ouib, and Mld. Séan and Ouib activate *nvd* and *spok* transcription via Séan and Ouib response elements in the *nvd* and *spok* promoters, respectively. To properly activate *nvd* and *spok* expression, Mld cooperatively interacts with Séan and Ouib.

In this study, we demonstrated that three ZAD-C_2_H_2_ zinc finger proteins, Séan, Ouib, and Mld, are required for ecdysone biosynthesis in the larval PG. The following points summarize our current findings in light of our previous studies (Danielsen *et al.* 2014; Komura-Kawa *et al.* 2015). First, loss-of-function mutations in *séan*, *ouib*, or *mld* severely reduced the expression of *nvd*, *spok*, or both *nvd* and *spok*, respectively. Second, we could rescue the larval arrest seen in animals without functional *séan*, *ouib*, and *mld* by supplementing intermediate metabolites of the ecdysone biosynthesis pathway in their diet. For example, the developmental arrest of *séan* mutants was restored by 7DC. Similarly, the larval arrest of *ouib* and *mld* mutants was restored by 5β-ketodiol supplementation. Third, the arrest of *séan*, *ouib*, or *mld* mutants was rescued by the PG-specific overexpression of *nvd* alone, *spo* alone (an ortholog of *spok*), or *nvd* and *spo* combined, respectively. Fourth, there are specific Séan and Ouib response elements in the *nvd* and *spok* promoter regions, respectively. Finally, the presence of Mld promotes synergistic action with Séan and Ouib to stimulate transcriptional upregulation. Based on these data, we propose that Séan, Ouib, and Mld act primarily to transcriptionally regulate just two Halloween genes, *nvd* and *spok*, in the PG (Figure 6**).**

Our *luc* reporter plasmid-based assay confirmed that Séan and Ouib, along with Mld, were sufficient to drive gene expression under the control of the *nvd* and *spok* promoters, respectively, in cultured S2 cells. However, we should point out that, in our current assay, the inducible activities of Séan and Ouib are considerably different. Although coexpression of *ouib* and *mld* exhibited a ∼400-fold induction of *luc* expression compared to that of controls, the induction caused by *séan* and *mld* was only 10-fold. Moreover, although coexpression of *ouib* and *mld* in S2 cells induced endogenous *spok* expression, coexpression of *séan* and *mld* did not induce endogenous *nvd* expression. Currently, it is unclear what causes this difference between Séan and Ouib. One possible explanation is that the difference might be caused by endogenous *séan* and *ouib* expression in S2 cells. We found that S2 cells used in this study expressed considerable amounts of *séan* and *mld*, but not *ouib* (Figure S8 in File S1). This implies that the *nvd* promoter region might be preloaded by endogenous Séan and Mld, and therefore that the overexpression of Séan and Mld may not achieve high induction of the *nvd* gene. Alternatively, Séan, but not Ouib, may require one or more indispensable TF(s) to sufficiently drive *nvd* expression. Along these lines, it would be interesting to test whether and how Séan and Mld cooperate with previously identified TFs that are necessary for *nvd* expression in the PG, including the CncC-dKeap1 complex (Deng and Kerppola 2013), Knirps (Danielsen *et al.* 2014), and Ventral veins lacking (Cheng *et al.* 2014; Danielsen *et al.* 2014).

It is currently difficult to completely rule out the possibility that Séan, Ouib, and Mld are involved in the direct transcriptional regulation of genes other than *nvd* and *spok*. While we found no DNA sequences that exactly matched the Séan-Mld response element (5′-AGCTTTATTGCTCAG-3′) elsewhere in the *D. melanogaster* genome, our previous study had found that some degenerate Ouib response elements exist in the genome, including the regions upstream of the coding regions of some Halloween genes (Komura-Kawa *et al.* 2015). Indeed, *séan* mutants show a significant elevation of other Halloween genes. However, we expect that these effects could be indirect because we also saw substantial upregulation of the same Halloween genes when we knocked down *nvd* alone via PG-specific RNAi. Thus, upregulation of these Halloween genes possibly reflects an attempt to compensate for low ecdysone production. To further clarify whether Séan and Ouib directly regulate other genes, additional studies will be required, such as chromatin immunoprecipitation sequencing analysis, together with an eventual mutational analysis of any identified targets.

Our RNA-Seq analysis also confirmed the upregulation of additional genes linked to ecdysteroid synthesis, including *Cyp18a1*, *sit*, *Start1*, *nobo*, and *ftz-f1*, in Séan-depleted ring gland samples (Table S2 in File S2). Consistent with this, we found genes encoding ecdysteroid-linked TFs that are classified as repressors, *Broad* and *knirps*, in the set of downregulated genes (Table S3 in File S2). One known repressor of ecdysone biosynthesis, HR4 (Ou *et al.* 2011), was markedly downregulated in Séan-depleted ring glands (Table S4 in File S2), raising the possibility that Séan regulates the *HR4* gene. Loss of *HR4* function in the PG is not lethal, consistent with the finding that *séan* mutants can be rescued by transgenic *nvd* expression. *HR4* depletion in the PG does not result in substantial upregulation of *sad* or *phm*, at least not in the early L3 stage (Ou *et al.* 2011), suggesting that other TFs are inducing the Halloween genes in *séan* mutants. The RNA-Seq data also revealed increased expression of genes with roles in cholesterol, heme, and ATP metabolism. One would expect these processes to sustain increased Halloween gene expression, since these enzymes need energy, metabolize cholesterol intermediates, and the P450 subset of ecdysone-producing enzymes has heme moieties as protein cofactors.

In this study, it is still unclear whether the three ZAD-C_2_H_2_ zinc finger proteins described here play roles in tissues other than the PG. Unlike *ouib*, which we can only detect in the PG (Komura-Kawa *et al.* 2015), we found that *séan* is highly expressed but not limited to the PG. This observation is partly consistent with the earlier finding that *séan* is expressed in the muscle founder cells of *D. melanogaster* embryos (Dobi *et al.* 2014). Interestingly, the Mld protein is also not limited to the PG but is also found in other tissues, including the imaginal discs, fat body, and the salivary gland during larval development (Neubueser *et al.* 2005). Further, the muscle morphology in *séan* mutants is disorganized in *D. melanogaster* embryos (Dobi *et al.* 2014), but no detailed functional studies have been reported. Thus, it is possible that *séan* and *mld* may have other functions that are not essential for viability but may be important for optimal fitness in the wild. On the other hand, *séan* and *mld* expression outside the PG raises the question as to why *nvd* or *spok* are not expressed in the non-PG tissues where Séan and Mld are present. One possibility is that these ZAD-C_2_H_2_ zinc finger proteins require other cofactors, which may be present in the PG, to induce *nvd* and/or *spok* expression. Alternatively, considering that most other identified ecdysteroidogenic TFs are not restricted to the PG either (Niwa and Niwa 2016a,b), one would expect that there are repressive mechanisms, such as chromatin states, that keep ecdysteroidogenic genes turned off in tissues other than the PG.

From an evolutionary point of view, any clear orthologs of *séan*, as well as *mld* and *ouib* (Neubueser *et al.* 2005; Ono *et al.* 2006; Komura-Kawa *et al.* 2015), are not found in any species thus far investigated other than Drosophilidae. Previous studies on *spok* hypothesized that the presence of *mld* and *ouib* might be coupled with the evolutionary appearance of *spok* in Drosophilidae, where duplication from the ancestral gene *spo* created the two paralogs (Ono *et al.* 2006; Komura-Kawa *et al.* 2015). However, our current study on *séan* suggests that this hypothesis may be untenable. This is because almost all insect genomes examined so far contain a single, unduplicated ortholog of *nvd*, placing the Mld/Séan/Ouib-*nvd*/*spo* axis outside the Drosophilidae (Yoshiyama *et al.* 2006; Yoshiyama-Yanagawa *et al.* 2011; Lang *et al.* 2012). Surprisingly, a gene synteny analysis suggests that *séan* orthologs are absent in some of the Drosophilidae species, such as *D. ananasse*, *D. virillis*, and *D. mojavensis* (Figure S4 in File S1). Such an evolutionary pattern raises the question as to which TFs regulate *nvd* expression in species that lack the *séan* or *mld* genes. It is noteworthy that *nvd* expression is spatiotemporally regulated in several lepidopteran species (Iga *et al.* 2013; Ogihara *et al.* 2015, 2017), the honeybee (Yamazaki *et al.* 2011), and even in a noninsect arthropod (Sumiya *et al.* 2016). It would be interesting to identify and characterize TFs responsible for *nvd* expression in these species. Individual insect genomes tend to harbor distinct sets of ZAD-C_2_H_2_ zinc finger genes, which expanded in an insect lineage-specific manner (Chung *et al.* 2007). Thus, it is possible that different ZAD-C_2_H_2_ zinc finger genes have evolved to regulate *nvd* expression in different species.

It should be noted that in *D. melanogaster*, both *nvd* and *spok* are located in the pericentromeric region of the third chromosome (Ono *et al.* 2006; Yoshiyama *et al.* 2006), which is thought to form constitutive heterochromatin (Fitzpatrick *et al.* 2005). Constitutive heterochromatin is a fundamental component of eukaryotic genomes and is believed to ensure a condensed and transcriptionally inert chromatin conformation in all cells of an organism [reviewed by Dimitri *et al.* (2009), Elgin and Reuter (2013), and Saksouk *et al.* (2015)]. Despite this, studies in the last four decades have revealed that constitutive heterochromatin contains active genes that are essential for viability in many organisms, including *D. melanogaster* [reviewed by Dimitri *et al.* (2009)] and mice (Rudert *et al.* 1995; Probst *et al.* 2010). To the best of our knowledge, Séan, Ouib, and Mld represent the first example of a set of TFs that regulate genes located in constitutive heterochromatin in a spatiotemporal-specific manner.

How do these three ZAD-C_2_H_2_ zinc finger proteins induce *nvd* and *spok* expression from a supposedly “inert” chromosomal region? In general, silenced heterochromatin displays distinctive chromatin characteristics including global hypoacetylation, trimethylation of histone H3 on lysine 9, and recruitment of the heterochromatin protein HP1 [reviewed by Elgin and Reuter (2013) and Timms *et al.* (2016)]. The ZAD domain is thought to serve as a protein–protein interaction domain (Jauch *et al.* 2003), suggesting that Séan and Ouib, along with Mld, might recruit cofactors that promote histone acetylation, followed by a reduction of H3K9m3 and reduced HP1 binding. Our analyses failed to detect any protein–protein interactions between Séan and Mld or between Ouib and Mld (T. Kamiyama and R. Niwa, unpublished results), suggesting that there are other interacting proteins, such as chromatin factors. Previous studies have reported that the zinc finger-containing proteins GAGA (Tsukiyama *et al.* 1994), Prod (Platero *et al.* 1998), and Su(var)3-7 (Cléard and Spierer 2001) associate with the heterochromatic chromocenter in *D. melanogaster*. Proteomic analysis to examine whether these or other factors physically interact with Séan, Ouib, and Mld are currently underway. These efforts will allow us to further elucidate the mechanisms by which heterochromatic gene expression is controlled.

## 

## Supplementary Material

Supplemental material is available online at www.genetics.org/lookup/suppl/doi:10.1534/genetics.117.300268/-/DC1.
