# Supplementary material for: Cooperative Control of Ecdysone Biosynthesis in Drosophila by Transcription Factors Séance, Ouija Board, and Molting Defective
Source: Genetics. 2017 Nov 29;208(2):605–22. doi: 10.1534/genetics.117.300268 (PMC5788525; doi:10.1534/genetics.117.300268)
Supplement: Supplementary file 1 [file 605FileS1.pdf]

Supplemental Figures

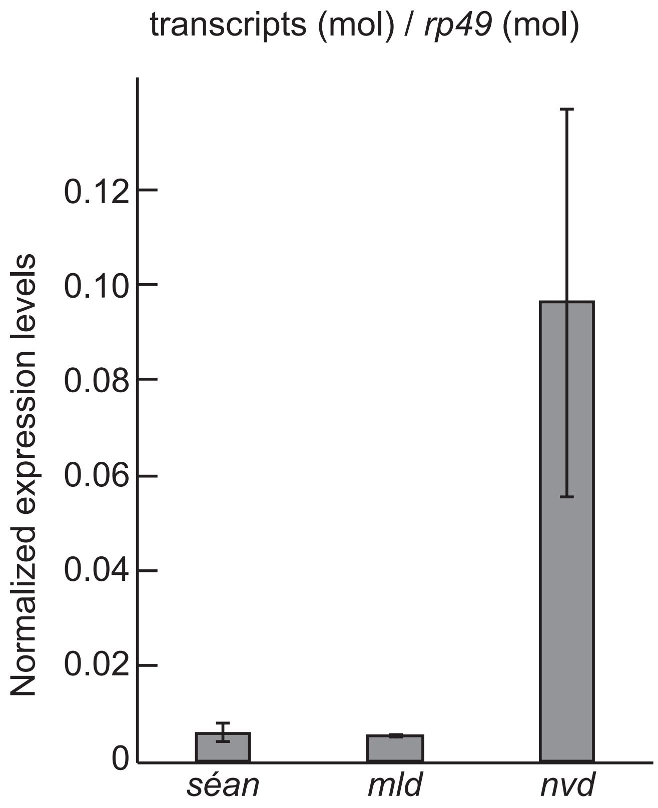

Figure S1. Expression levels of *séan*, *mld*, and *nvd* in the adult ovaries. Expression levels (the number of moles of transcripts) were normalized by the number of moles of *rp49* transcripts, each at N=3. Error bars are s. e. m. Neither *séan* nor *mld* transcripts in the ovary were at low levels. In contrast, the higher level of *nvd* transcripts was detected in the ovary, consistent with the previous study (Yoshiyama et al. 2006).

```
wt          MLINVCRVCGRSRLCPKAVELFKPGRQDILRRIQLITGILLQQIPNAPDMVCFCCQTDLQ
séance557   MLINVCRVCGRSRLCPKAVELFKPGRQDILRRIQLITGILLQQIPNAPDMVCFCCQTDLQ

wt          SAMIFRRQCILQQKKWVPLLQSDKVGASEEKKVEPNDPSTKKKTTKRRRGRPRMPLEIVD
séance557   SAMIFRRQCILQQKKWVPLLQSDKVGASEEKKVEPNDPSTKKKTTKRRRGRPRMPLEIVD

wt          IVVTNESKASAGESVGGDEFDQPVEISNEPDATDSDVNLEEIDLPDEDGLESDHDLPNVQ
séance557   IVVTNESKASAGESVGGDEFDQPVEISNEPDATDSDVNLEEIDLPDEDGLESDHDLPTIS

                         1                                 2
wt          IHKCDTCGIIKNNKSSLVRHQFEHNGIRPYPCK----ECPKTFLVASELKAHNLTHHTLE
séance557   SYLVHYHDLISIHCAYIHTYVCKFFFFVQYLQYVFFRECPKTFLVASELKAHNLTHHTLE

                         3                               4
wt          PPFACRYCDRRYFSVVGRKKHERVHTNERPFVCDQCGKAFTRTCILKAHMAVHQVVRKYS
séance557   PPFACRYCDRRYFSVVGRKKHERVHTNERPFVCDQCGKAFTRTCILKAHMAVHQVVRKYS

                    5
wt          CDVCDRSFSLKKHLATHFISNTHKRNAEAVTSSSEYMSMLSFESDETWSQGTPLTTSIDE
séance557   CDVCDRSFSLKKHLATHFISNTHKRNAEAVTSSSEYMSMLSFESDETWSQGTPLTTSIDE

wt          DLVQSQFDLLCQEV 370
séance557   DLVQSQFDLLCQEV 374
```

Figure S2. Alignment of the predicted amino acid sequences of wild type (wt) *séance*, and the *séance*^{557} alleles. Green: ZAD domain. Brackets with circled numbers correspond to the five C_2H_2 zinc finger domains (cysteine and histidine residues are shown in yellow). The *séance*^{557} allele is the result of a 171 bp deletion that removes 104 bp from the 3′ end of exon #2 and 67 bp from the 5′ end of intron #2. This causes a predicted in-frame read-through of the remaining intron #2 sequence, resulting in a stretch of 41 inappropriate amino acids (shown in red) that are incorporated into the *séance*^{557}-encoded protein, removing zinc finger domain #1 entirely and the first cysteine of zinc finger domain #2. Results are based on sequencing of the genomic *séance*^{557} allele, not actual cDNA.

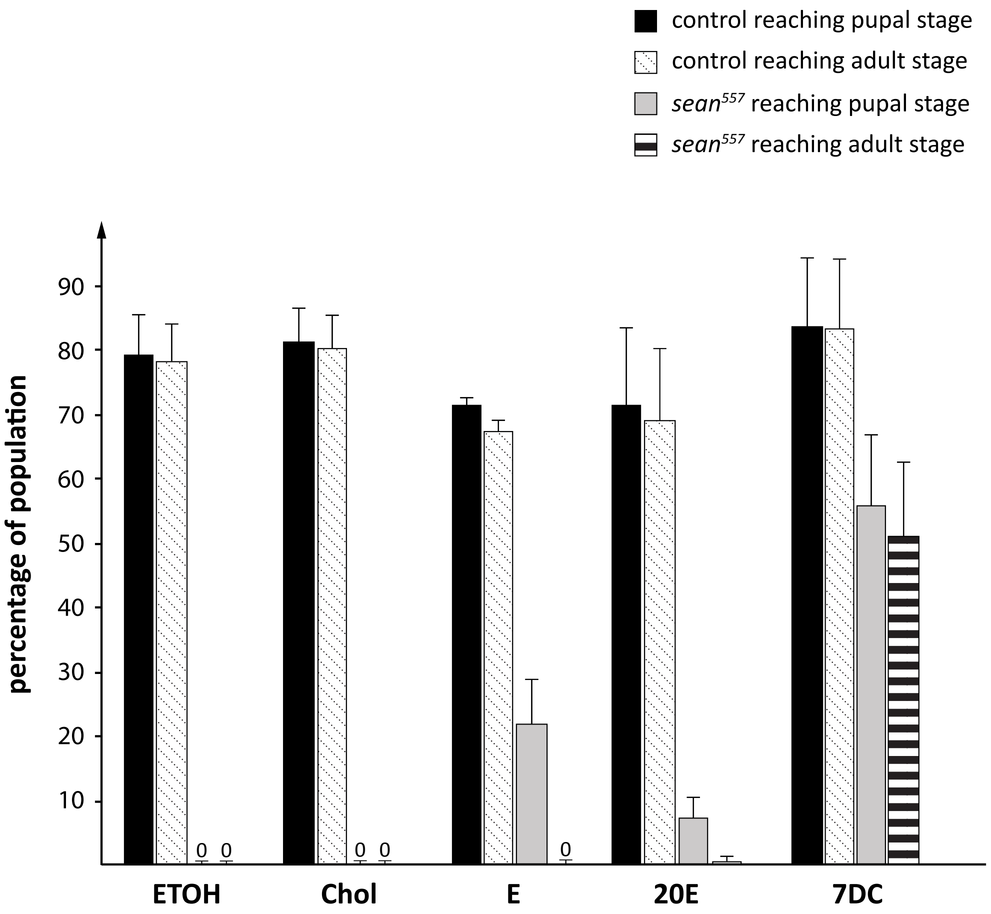

Figure S3. Dietary sterol supplementation of *séance*[557] mutants. Larvae were fed diets containing either ethanol (ETOH, acts as a carrier control), cholesterol (Chol), α-ecdysone (E), 20-hydroxyecdysone (20E), or 7-Dehydrocholesterol (7DC). Only administration of 7DC yielded significant survival to adulthood, but both E and 20E led to a partial rescue, allowing mutants to develop to pupal stages.

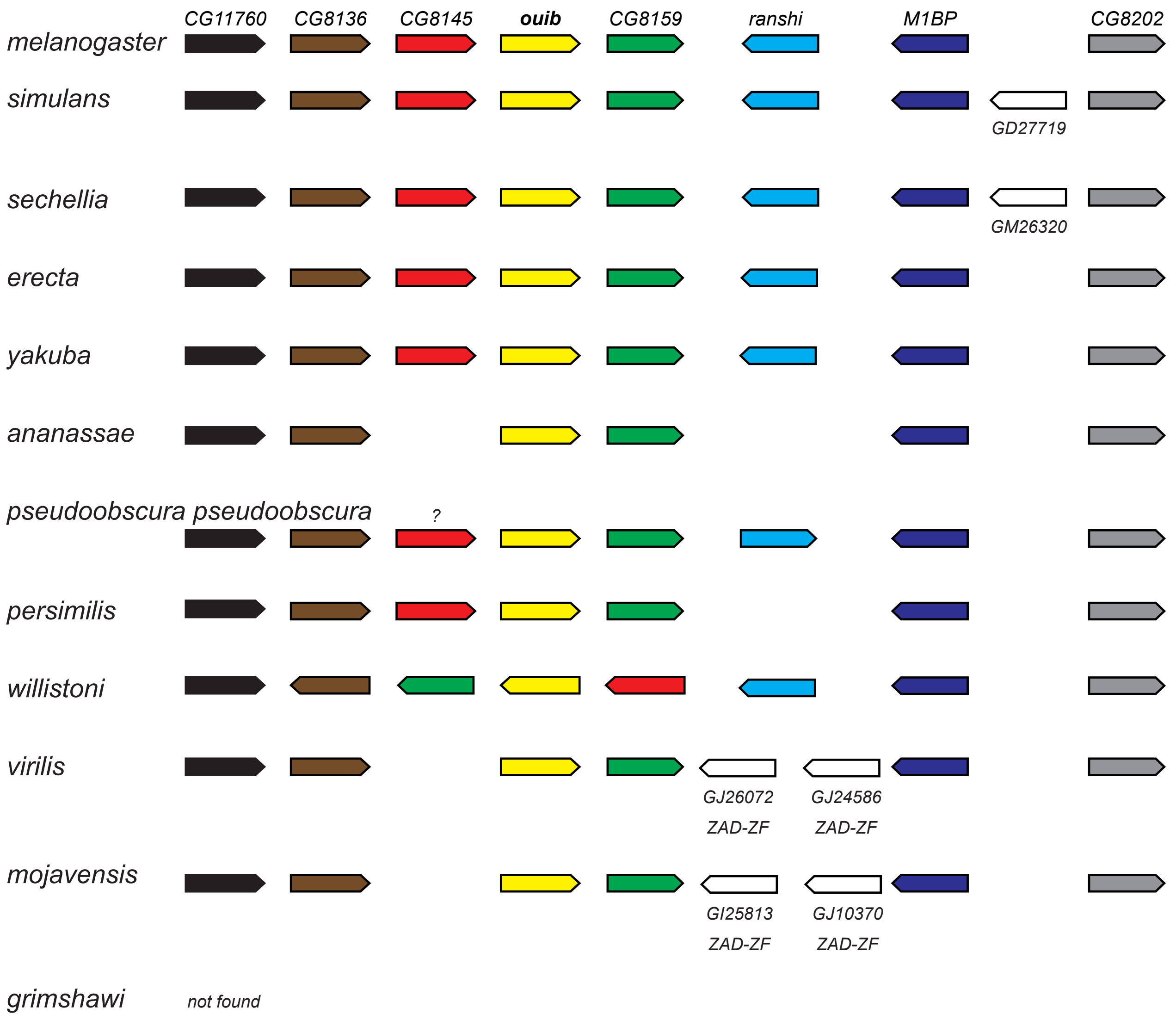

Figure S4. Synteny of *séan*, *ouib*, and other surrounding genes in Drosophilidae species. Genome information was obtained from FlyBase. Each gene is represented by an arrow-like box, whose arrowhead indicates the 3′ direction of the gene. Séan (CG8145), *ouib*, *CG8159*, *ranshi*, and *M1BP* are all ZAD-C_2H_2 zinc finger genes, whereas *CG11760*, *CG8136*, and *CG8202*, *GD27719*, and *CG26320* are not. Each ortholog is represented by the same color, except for genes represented by white, which are not orthologous to each other. *D. grimshawi* genome information around these loci was not available. Note that some species lack clear orthologs of *séan* (*CG8145*).

melanogaster	−283	AGCTTTATTGCTCAG	−269	
simulans	−111	AGCTTTATTGCTCAG	−97	(15/15)
sechellia	−213	AGCTTTATTGCTCAG	−209	(15/15)
willistoni	−137	ATGTAAATTGATCAG	−123	(10/15)

Figure S5. The evolutionary conservation of sequences similar to Séan-response element in putative *nvd* enhancer/promoter regions of some Drosophilidae species. EMBOSS Matcher (McWilliam et al., 2013) was used to search for sequences similar to the *D. melanogaster* Séan-response element (15 bp) within the 2 kb regions upstream of the translation initiation site of the *nvd* loci from *D. simulans* (GD11948), *D. sechellia* (GM18285), and *D. willistoni* (GK11941). Numbers before and after nucleotide sequences indicate the distance from the translation initiation site of *nvd*. Parentheses indicate numbers of identical matches to *D. melanogaster* Séan-response element. Note that genome sequence data around *nvd* loci from other *Drosophila* species were not available.

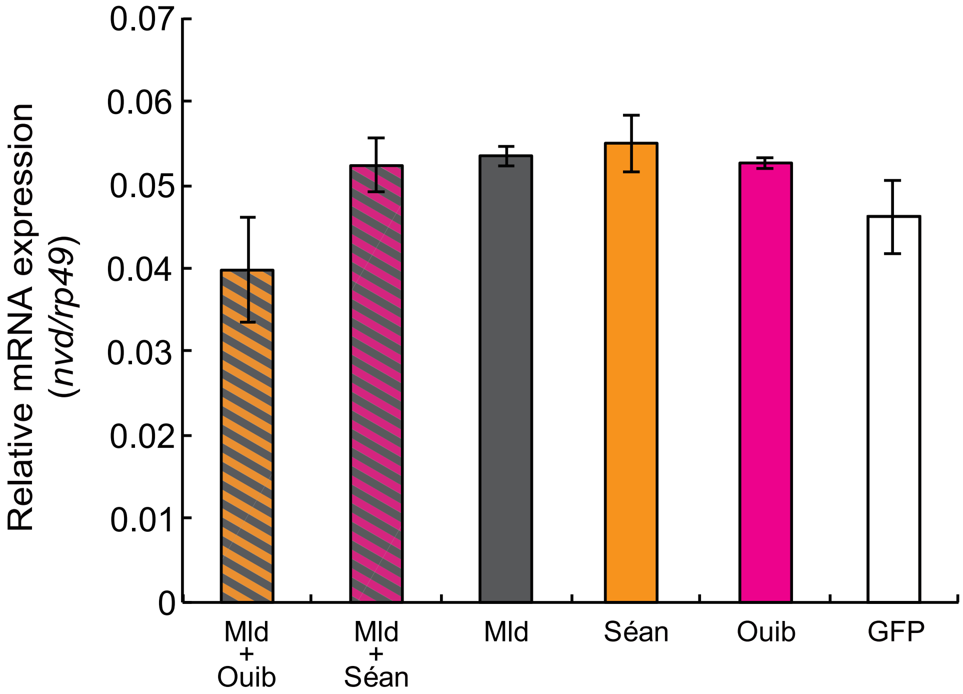

Figure S6. qPCR analysis to determine endogenous *neverland* expression levels in S2 cells transfected with different expression constructs.

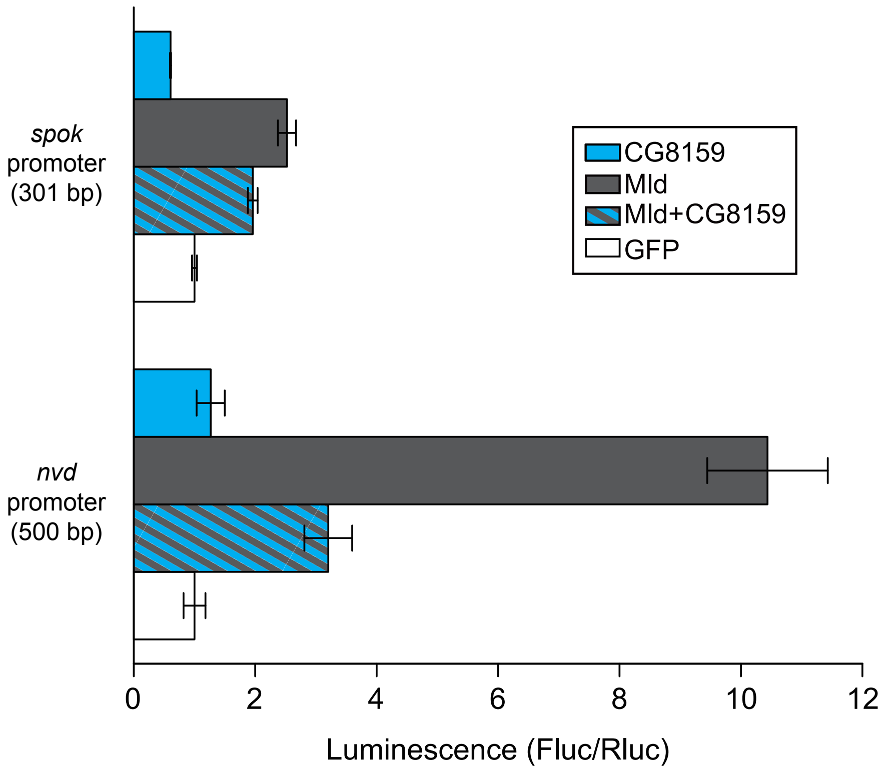

Figure S7. Luciferase reporter assay with *CG8159* and/or *mld* expression plasmids and *luc* plasmids. The *luc* plasmids contain the upstream elements of *spok* (the +331 to +32 bp region; 301 bp) and *nvd* (the +500 to +1 bp region; 500 bp). The GFP expression plasmid was used as a negative control, each at N=3.

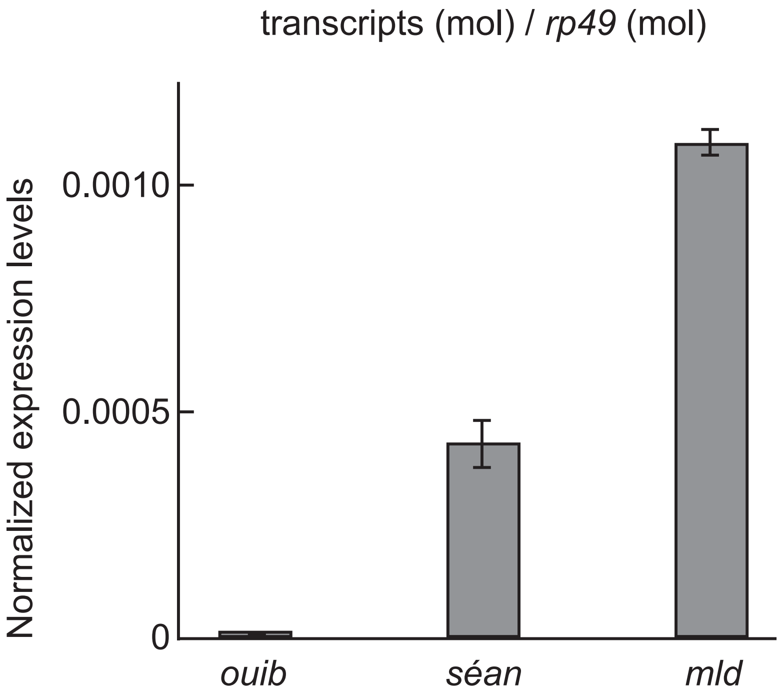

Figure S8. Expression levels of *ouib*, *séan*, and *mld* in S2 cells used in this study. Expression levels (the number of moles of transcripts) were normalized by the number of moles of *rp49* transcripts, each at N=3. Error bars are s. e. m.
