# Supplementary material for: Cooperative Control of Ecdysone Biosynthesis in Drosophila by Transcription Factors Séance, Ouija Board, and Molting Defective
Source: Genetics. 2017 Nov 29;208(2):605–22. doi: 10.1534/genetics.117.300268 (PMC5788525; doi:10.1534/genetics.117.300268)
Supplement: Supplementary file 2 [file 605FileS2.pdf]

Table S1. Oligonucleotides used in this study.

Purpose	Name of primer	Sequence (5´-3´)
Cloning of *séan* CDS	CG8145_CDS_F	ATGTTAATAAATGTGTGCCGAGTTTGCG
	CG8145_CDS_R	TTAAACTTCCTGACACAAAAGGTCGAATTGC
qRT-PCR primers for amplifying *séan* (for Figures S1 and S8)	CG8145_qRT-PCR_NEW_F	TACCCAATGTTCAAATCCACAAGTGCGATACTTGTGG
	CG8145_qRT-PCR_NEW_R	CGCTACCAGAAAGGTCTTTGGGCACTCC
qRT-PCR primers for amplifying *mld* (for Figures S1 and S8)	mld_qPCR_Fwd	CGAGGAGATGACCATTTGGATGC
	mld_qPCR_Rev	TTTCCGCCGGAACTTGGCCAC
Real-time PCR (for analyses on *séan*^{557} allele and *séan*-RNAi animals in Figure 3C, and all data in Figure 3D)	rp49_qPCR_F	CGGATCGATATGCTAAGCTGT
	rp49_qPCR_R	GCGCTTGTTCGATCCGTA
	Séance_qPCR_F	CCGTGGAGCTCTTCAAACC
	Séance_qPCR_R	CATTGGGAATCTGCTGAAGG
	Neverland_qPCR_F	CCCTCACCTAGGAGCCAACT
	Neverland_qPCR_R	GGCATATAACACAGTCGTCAGC
	shroud_qPCR_F	CGAATCGCTGCACATGAC
	shroud_qPCR_R	TAGGCCCTGCAGCAGTTTAG
	spookier_qPCR_F	GCGGTGATCGAAACAACTC
	spookier_qPCR_R	CGAGCTAAATTTCTCCGCTTT
	phantom_qPCR_F	GGCATCATGGGTGGATTT
	phantom_qPCR_R	CAAGGCCTTTAGCCAATCG
	diembodied_qPCR_F	GTGACCAAGGAGTTCATTAGATTTC
	diembodied_qPCR_R	CCAAAGGTAAGCAAACAGGTTAAT
	shadow_qPCR_F	CAAGCGGATATTTGTAGACTTGG
	shadow_qPCR_R	AAGCCCACTGACTGCTGAAT
Vector construction for CRISPR/Cas9 system	CG8145_CRISPR_oligo3_s	cttcGCCGTGGAGCTCTTCAAACC
	CG8145_CRISPR_oligo3_as	aaacGGTTTGAAGAGCTCCACGGC
	CG8145_CRISPR_oligo6_s	cttcGATTCCCAATGCTCCGGATA
	CG8145_CRISPR_oligo6_as	aaacTATCCGGAGCATTGGGAATC
	CG8145_CRISPR_oligo_8	TACAAGTTTAGGTGTTCACGTGG
	CG8145_CRISPR_oligo_9	CCACGTGAACACCTAAACTTGTA
Amplifying the target sites of CRISPR/Cas9 system	CRISPR_8145_check_F	AACTTAGCGGGTAATTTCAAATCTAATGTGCC
	CRISPR_8145_check_R	CTTCGCCGCTTGGTCGTTTTC
Sequencing *séan*^{557} locus	seanceS_F	TGCAGCAGAAGAAATGGGTG
	seanceS_R	TCGCAACTAAAACGGGAATGA
Construction for pWALIUM10-moe with V5 tags at N-terminus of an insert	pWAL-V5-N-F	AATTATGGGCAAGCCCATCCCCAACCCCCTGCTGGGCCTGGATTCCACCA
	pWAL-V5-N-R	GATCTGGTGGAATCCAGGCCCAGCAGGGGGTTGGGGATGGGCTTGCCCAT
Vector construction to overexpress *V5-séan*	CG8145_N-V5_F	catatggcATGTTAATAAATGTGTGCCGAGTTTGCG
	CG8145_N-V5_R	gctagcTTAAACTTCCTGACACAAAAGGTCGAATTGC

Vector construction to overexpress *HA-mld*	mld_gw_Fwd	caccATGAGTGCCAACCGAAGAAGACG
	mld_gw_Rev_stop	TTATGGCGTTGTGGCATAGGATTCC
Vector construction to overexpress a *D. melanogaster nvd* cDNA<br><br>(Mutated bases are underlined.)	EXON1FOR	GGATGTCATTGCTAAAGAATAATTGGAAACCGATCAGTAATG
	EXON1REV	CATTACTGATCGGTTTCCAATTATTCTTTAGCAATGACATCC
	EXON2FOR	GCTGACGACTGTGTTATATGCCCTTTTCATCAATGG
	EXON2REV	CCATTGATGAAAAGGGCATATAACACAGTCGTCAGC3
Construction of 3xTy1-tag pBluescript II SK(-)	Ty1_x3_For	GAGGTGCACACCAACCAGGACCCCCTGGACGCCGAAGTCCATACAAATCAGGATCCTCTGGATGCCGAAGTGCACACCAATCAGGATCCCCTGGACGCT
	Ty1_x3_Re	AGCGTCCAGGGGATCCTGATTGGTGTGCACTTCGGCATCCAGAGGATCCTGATTTGTATGGACTTCGGCGTCCAGGGGGTCCTGGTTGGTGTGCACCTC
Amplifying Ty1-tag fragment for *Ty1-CG8159* construction	Ty1ForprimerpBlueGib	GAATTCCTGCAGCCCATGGAGGTGCACACCAACCAGG
	Ty1-Rev-primer	AGCGTCCAGGGGATCCTG
Amplifying CG8159 coding region for *Ty1-CG8159* construction	CG8159-Fwd-Gib	GGATCCCCTGGACGCTATGCTGCAAAATGTGTGCCG
	CG8159-Rev-Gib	ACTAGTGGATCCCCCCTAGTTTGTACTATACATTAGAG
nvd-luciferase vector construction<br><br>(Lower case letters indicate restriction enzyme recognition sites.)	nvd_upstream_5kb_F	gcggccgcAGCCTAAATCCTAAAAAACTG
	nvd_upstream_4kb_F	gcggccgcATTTGGCCCGAACCC
	nvd_upstream_3kb_F	gcggccgcATCTTGTAAAAGTATCAGTGAAAAAG
	nvd_upstream_2kb_F	gcggccgcGTATGAGAAAAACGAGCG
	nvd_upstream_1kb_F	gcggccgcATTCCGCTCGTGAAAG
	nvd_upstream_900b_F	gcggccgcTAAAAAACCGCAACCACATA
	nvd_upstream_800b_F	gcggccgcGTAGTGCTCAAATCAGGCAC
	nvd_upstream_700b_F	gcggccgcGTATAATATGTAATATGTCGCTGTAAC
	nvd_upstream_600b_F	gcggccgcAATTATGATATATATTAATTTTTTTAGTC
	nvd_upstream_500b_F	gcggccgcTATTATGGATTATAAAGATACATAAATATAAACAGGTAATATACATATTTTC
	nvd_upstream_301b_F	gcggccgcGCCGGAAAAATATTAAATAGC
	nvd_upstream_281b_F	gcggccgcCTTTATTGCTCAGCTAAGGACCAC
	nvd_upstream_261b_F	gcggccgcCCACCCTAACGAAAACGGTGAAC
	nvd_upstream_R1	agatctATTATTAATAAGTATCTATAATAATAG

nvd-luciferase construct with transversion mutation<br><br>(Underline indicates the transversion mutation sequences.)	nvd_upstream_300b ver_F1	ATAAGAATGCGGCCGCGCCGGAAAAATATTAAATCTAGGGCGGTAGACTCTAAGGACC
	nvd_upstream_R2	GGAAGATCTATTATTAATAAGTATCTATAATAATAG
spok-luciferase vector construction<br><br>(Lower case letters indicate restriction enzyme recognition sites.)	spok_upstream_111-32b F	actgagctcTCTTTTTTATAGAATTTATT
	spok_upstream_111-32b R	ggaagatctCTTATATACAAAAACCCTTT
	spok_upstream_91-32b F	cTTAAATATGCGTTTGACCTATGAAATTTTATTGTTGTTGGAAAGGGTTTTTGTATATAAGa
	spok_upstream_91-32b R	gatctCTTATATACAAAAACCCTTTCCAACAACAATAAAATTTCATAGGTCAAACGCATATTTAAgagct
	spok_upstream_71-32b F	cTGAAATTTTATTGTTGTTGGAAAGGGTTTTTGTATATAAGa
	spok_upstream_71-32b R	gatctCTTATATACAAAAACCCTTTCCAACAACAATAAAATTTCAgagct

Table S2: GO term ("Biological Process") enrichment for genes with differential expression in *séance*-RNAi prothoracic glands (360 upregulated genes).

GO Term	GO Term Description	Count	*P* Value	Genes	Fold Enrichment
0055114	oxidation-reduction	29	9.4E-07	*Trxr-1 Aldh Cyp317a1 P5CDh1 phm sad AOX3 Cyp6a13 Cyp4g1 CG9747 CG32557 Hmgcr spok Fmo-2 CG9512 CG3719 FASN2 Jafrac1 Cyp18a1 CG9674 Gpdh CG15093 Sod1 Nmdmc Cyt-b5 CG5167 Cyp6a9 CG17896 Drat*	2.9
0006497	protein lipidation	4	1.1E-03	*l(2)efl por stv CG14207*	18.1
0006117	acetaldehyde metabolic process	3	5.7E-03	*Aldh Adh AOX3*	24.4
0006694	steroid biosynthesis	3	8.4E-03	*CG17597 sit Start1*	20.4
0035074	pupation	3	1.2E-02	*Ude ftz-f1 Cyp18a1*	17.5
0018990	ecdysis, chitin-based cuticle	4	1.4E-02	*Idgf3 Cht7 Idgf4 Hmgcr*	7.8
0006032	chitin catabolism	4	2.2E-02	*Idgf3 Idgf2 Cht7 Idgf4*	6.5
0008340	determination of adult lifespan	10	2.4E-02	*Trxr-1 Tsp42Ef AGBE VhaSFD cher CG30427 Jafrac1 Sod1 Trx-2 ImpL2*	2.4
0006749	glutathione metabolism	5	2.7E-02	*GstS1 GstD10 GstT4 GstD9 GstE14/nobo*	4.3
0030431	sleep	8	3.0E-02	*l(3)02640 Tsp97E cher tim Cpr67B Acer Drat Tsp42El*	2.7
0042632	cholesterol homeostasis	3	3.3E-02	*mag sit GstE14/nobo*	10.2
0035556	intracellular signal transduction	7	3.7E-02	*Sbf CG4325 CG7510 Sik2 CG10737 dop Plc21C*	2.8
0006697	ecdysone biosynthesis	3	3.9E-02	*spok sad phm*	9.4
0046034	ATP metabolism	3	4.5E-02	*Adk1 scu CG17726*	8.7
0006090	pyruvate metabolism	3	4.5E-02	*Aldh Pdp l(1)G0334*	8.7
0006783	heme biosynthesis	3	4.5E-02	*Pbgs FeCH CG34423*	8.7

Red: genes with known roles in cholesterol and ecdysone homeostasis.

Table S3: GO term ("Biological Process") enrichment for genes with differential expression in *séance*-RNAi prothoracic glands (248 downregulated genes).

GO Term	GO Term description	Count	*P* Value	Genes	Fold Enrichment
0030246	carbohydrate binding	11	7.4E-04	*Muc11A verm obst-A CG12009 Cht5 pgant4 LManII lectin-24Db PGRP-SC2 serp Cht10*	3.68
0019842	vitamin binding	8	8.4E-04	*apolpp CG1544 GlcAT-P CG5958 CG2663 Sply Oat pinta*	5.16
0004866	endopeptidase inhibitor	7	1.5E-03	*CG15369 CG16704 Reck Spn88Eb Spn43Aa CG32354 CG31777*	5.59
0030414	peptidase inhibitor	7	1.8E-03	*CG15369 CG16704 Reck Spn88Eb Spn43Aa CG32354 CG31777*	5.39
0001871	pattern binding	8	3.1E-03	*Muc11A verm obst-A CG12009 Cht5 PGRP-SC2 serp Cht10*	4.12
0030247	polysaccharide binding	8	3.1E-03	*Muc11A verm obst-A CG12009 Cht5 PGRP-SC2 serp Cht10*	4.12
0008061	chitin binding	7	4.5E-03	*Muc11A verm obst-A CG12009 Cht5 serp Cht10*	4.47
0005198	structural molecule	16	4.5E-03	*Eig71Ee Act87E Cpr78E Act42A Jupiter Sgs3 apolpp Sgs5 scb Sgs4 obst-A Sgs7 Sgs8 Cpr57A Arpc3B Cpr49Ac*	2.24
0005501	retinoid binding	3	6.3E-03	*apolpp CG5958 pinta*	23.95
0004857	enzyme inhibitor	7	6.6E-03	*CG15369 CG16704 Reck Spn88Eb Spn43Aa CG32354 CG31777*	4.14
0016564	transcription repressor	7	6.9E-03	*kni br E(spl)mbeta-HLH esg E(spl)m3-HLH ovo jing*	4.10
0019840	isoprenoid binding	3	9.9E-03	*apolpp CG5958 pinta*	19.16
0005344	oxygen transporter	3	1.7E-02	*Lsp2 glob1 Fbp1*	14.74
0003700	transcription factor	13	1.7E-02	*kni br peb scro E(spl)mbeta-HLH srp bab2 tin Eip75B knrl pnr E(spl)m3-HLH jing*	2.13
0016566	specific transcriptional repressor	4	1.8E-02	*kni br E(spl)mbeta-HLH E(spl)m3-HLH*	7.09
0004867	serine-type endopeptidase inhibitor	5	1.8E-02	*CG16704 Reck Spn88Eb Spn43Aa CG31777*	4.91
0008092	cytoskeletal protein binding	9	2.0E-02	*Hsp23 ena apolpp nuf Tektin-C sn Arpc3B Jupiter CG14687*	2.64
0005200	structural constituent of cytoskeleton	4	2.7E-02	*Act87E Act42A Arpc3B Jupiter*	6.08
0004099	chitin deacetylase	2	3.1E-02	*verm serp*	63.85
0004879	ligand-dependent nuclear receptor	3	4.9E-02	*kni Eip75B knrl*	8.33

Red: genes with known roles in cholesterol and ecdysone homeostasis.

Table S4. Fold changes for genes listed in Table 1.

Flybase IDs					linear total RPKM	
**FBgn**	**CG**	**SYMBOL**	**Fold Change**	**P value**	**Control**	**CG8145 RNAi**
FBgn0264562	*CG43934*	*Hr4*	-34.77	5.00E-02	14.19	0.41
FBgn0259697	*CG40050*	***nvd***	**-9.50**	4.24E-02	29.88	3.15
FBgn0032405	*CG14946*	*CG14946*	-8.98	9.98E-02	5.02	0.56
FBgn0014469	*CG2060*	*Cyp4e2*	-7.53	1.20E-01	6.12	0.81
FBgn0000568	*CG8127*	*Eip75B*	-4.07	1.07E-01	47.37	11.65
FBgn0001320	*CG4717*	*kni*	-3.84	6.63E-02	27.29	7.11
FBgn0001323	*CG4761*	*knrl*	-3.47	7.45E-02	25.83	7.44
FBgn0004959	*CG6578*	***phm***	**3.00**	1.07E-01	466.14	1399.22
FBgn0038038	*CG5167*	*CG5167*	3.03	1.47E-01	2.95	8.95
FBgn0030484	*CG1681*	*GstT4*	3.05	6.69E-02	1.90	5.79
FBgn0037138	*CG7145*	*P5CDh1*	3.05	1.13E-01	4.92	15.00
FBgn0003312	*CG14728*	***sad***	**3.13**	4.88E-02	2262.16	7070.60
FBgn0030593	*CG9512*	*CG9512*	3.28	1.96E-01	0.62	2.03
FBgn0040070	*CG31884*	*Trx-2*	3.44	2.49E-01	27.59	94.86
FBgn0086917	*CG41624*	***spok***	**3.89**	9.82E-02	464.55	1807.63
FBgn0001078	*CG4059*	*ftz-f1*	4.79	9.80E-03	0.84	4.03
FBgn0031360	*CG31937*	*CG31937*	4.96	1.81E-01	6.17	30.61
FBgn0024986	*CG3719*	*CG3719*	5.01	1.25E-01	1.89	9.44
FBgn0033304	*CG2397*	*Cyp6a13*	5.19	2.00E-02	0.65	3.36
FBgn0038349	*CG6045*	*AOX3*	5.75	3.35E-02	0.87	4.98
FBgn0010019	*CG3972*	*Cyp4g1*	6.41	9.64E-02	91.32	585.30
FBgn0038610	*CG7675*	*CG7675*	6.66	2.36E-02	10.11	67.32
FBgn0038020	*CG10091*	*GstD9*	7.08	1.06E-01	1.66	11.75
FBgn0010226	*CG8938*	*GstS1*	7.18	6.00E-02	41.22	296.14
FBgn0033817	*CG4688*	*GstE14/nobo*	7.53	1.86E-02	44.09	331.77
FBgn0033188	*CG1600*	*Drat*	7.88	2.68E-02	3.29	25.92
FBgn0013771	*CG10246*	*Cyp6a9*	8.18	1.30E-01	0.30	2.49
FBgn0052557	*CG32557*	*CG32557*	9.25	5.40E-03	81.53	754.09
FBgn0042627	*CG3524*	*v(2)k05816*	15.74	5.81E-02	0.29	4.54
FBgn0033982	*CG17453*	*Cyp317a1*	20.02	1.36E-01	0.13	2.65
FBgn0010383	*CG6816*	*Cyp18a1*	31.60	4.35E-02	0.15	4.65
FBgn0042206	*CG18548*	*GstD10*	49.89	3.52E-03	0.15	7.65

Halloween genes are indicated in blue (upregulated) and red (downregulated).

Table S5. Feeding rescue activity of 7DC and 20E for loss-of-function animals of *séance*, *neverland*, *noppera-bo*, and *Niemann-Pick type C lysosome storage disease gene a*.

Data were obtained by this study and from the published literature. All loss-of-function animals reared on normal food died during larval stages. Values in the Table represent percentages of animals that reached the adult stage when 7DC or 20E was supplemented in the food.

Genotype	7DC (%)	20E (%)	References
séan^{33}/*séan*^{60}	65	0	This study
phm>*nobo*-RNAi	81	24	Enya et al. 2014
phm>*nvd*-RNAi	97	4	Yoshiyama et al. 2006
npc1a/*npc1a*	30	0	Huang et al. 2005
